# Multiscale physical effects of CpG methylation on DNA mechanics, nucleosome wrapping, and chromatin condensates

**DOI:** 10.1038/s44319-026-00894-2

**Published:** 2026-09-03

**Authors:** Luuk J C Daris, Aoon Rizvi, Ashfeen Nawar, Anurag Singh, Joshua Hutchings, Lieke A Lamers, Elizabeth A Montabana, Dari Kimanius, Priya R Banerjee, Jorine M Eeftens, Serena Sanulli

**Affiliations:** 1 Department of Cell Biology, Radboud Institute for Molecular Life Sciences, Radboud University, Nijmegen, The Netherlands; 2 Department of Genetics, Stanford University, Stanford, CA USA; 3 Department of Physics, The State University of New York at Buffalo, Buffalo, NY USA; 4 Biohub, Redwood City, CA USA; 5 Department of Molecular Biology, Radboud Institute for Molecular Life Sciences, Oncode Institute, Radboud University, Nijmegen, The Netherlands; 6 Department of Biological Sciences, The State University of New York at Buffalo, Buffalo, NY USA

**Keywords:** Chromatin, Transcription & Genomics

## Abstract

DNA methylation at CpG dinucleotides is a central epigenetic modification linked to transcriptional repression and heterochromatin, yet how it contributes to chromatin compaction beyond recruiting methyl-binding proteins remains unclear. Here, we integrate single-molecule force spectroscopy, FRAP, and nanorheology to show that CpG methylation alters chromatin organization across scales. CpG methylation modestly stiffens and extends DNA, while weakening both outer- and inner- turn nucleosome DNA wrapping. Despite these local destabilizing effects and similar thresholds for salt-induced phase separation, methylated chromatin condensates exhibit reduced internal mobility and increased viscoelasticity, indicating denser internal organization. Finally, CpG methylation cooperates with H3K9me3 to promote HP1α–mediated chromatin condensation, despite not altering HP1α affinity for naked DNA. These findings indicate that HP1α does not sense CpG methylation through direct recognition of the DNA modification itself, but rather through methylation-dependent changes in chromatin architecture and material properties. Together, our results support a multiscale physical model in which CpG methylation contributes to heterochromatin organization by tuning DNA rigidity, nucleosome wrapping energetics, and condensate material properties.

## Synopsis

CpG DNA methylation reshapes chromatin across scales by altering DNA mechanics, nucleosome wrapping, and chromatin condensate material properties, revealing a physical mechanism by which DNA methylation contributes to heterochromatin organization.

- CpG methylation alters DNA and nucleosome mechanics by increasing DNA stiffness while weakening nucleosome wrapping, reshaping the energetic landscape of nucleosome assembly and DNA breathing.
- These local mechanical changes propagate across scales to generate more viscoelastic, dynamically constrained chromatin condensates with enhanced internucleosome connectivity.
- CpG methylation cooperates with H3K9me3 to promote HP1α-mediated chromatin condensation, providing a physical mechanism linking DNA methylation to heterochromatin organization.

## Abstract (web summary)

CpG DNA methylation reshapes chromatin across scales by altering DNA mechanics, nucleosome wrapping, and chromatin condensate material properties, revealing a physical mechanism by which DNA methylation contributes to heterochromatin organization.

## Graphical abstract

**Figure:**
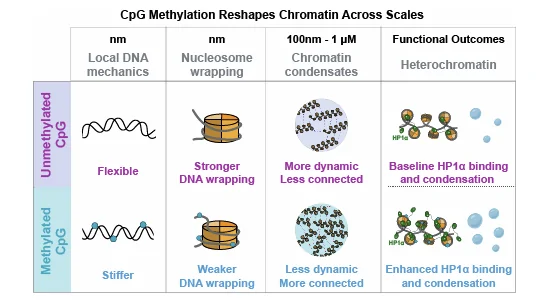

## Introduction

The eukaryotic genome is packaged within the confined volume of the nucleus as chromatin, forming hierarchically organized structures that span multiple length scales (Gibcus and Dekker, 2013; Finn and Misteli, 2019). The first level of chromatin organization involves the wrapping of DNA around an octamer of histone proteins to form nucleosomes, the basic repeating units of chromatin (Richmond et al, 1984; McGinty and Tan, 2015). Arrays of nucleosomes fold further into higher-order structures, including chromatin fibers, topologically associated domains, and chromosome-scale compartments (Gibcus and Dekker, 2013; Finn and Misteli, 2019). Beyond enabling efficient genome packaging, chromatin folding functionally organizes the genome to regulate genome processes, such as transcription, replication, and gene silencing (Misteli, 2020; Batut et al, 2022).

Chromatin exists along a continuum of compaction states that broadly correlate with transcriptional states (Ou et al, 2017; Li et al, 2021; Ricci et al, 2015; Nozaki et al, 2017). Euchromatin, which is relatively open and accessible, is associated with active transcription, permissive histone modifications, and dynamic chromatin-binding factors (Minami et al, 2025). In contrast, heterochromatin represents a more compact, transcriptionally repressive state that plays essential roles in genome stability, developmental gene silencing, and nuclear organization (Minami et al, 2025; Allshire and Madhani, 2018). Heterochromatin domains can form constitutively at repetitive and pericentromeric regions, or facultatively in a cell-type-specific and developmentally regulated manner (Saksouk et al, 2015; Grewal, 2023; Nicetto and Zaret, 2019). These compact domains not only restrict transcriptional access but also serve as structural scaffolds involved in nuclear organization (Allshire and Madhani, 2018; Janssen et al, 2018; Stephens et al, 2019).

At the molecular level, heterochromatin is characterized by specific biochemical hallmarks. Among the most conserved and functionally linked are histone H3 lysine 9 trimethylation (H3K9me3) and DNA methylation (Allshire and Madhani, 2018; Sanulli et al, 2019a; Du et al, 2015; Cedar and Bergman, 2009). H3K9me3 is recognized by heterochromatin protein 1α (HP1α), which binds nucleosomes, promotes chromatin compaction in condensates, and drives the formation of microscopically visible heterochromatic foci (Allshire and Madhani, 2018; Sanulli et al, 2019a; Keenen et al, 2021; Larson et al, 2017; Sanulli et al, 2019b). DNA methylation at CpG dinucleotides similarly contributes to gene repression by recruiting methyl-CpG-binding proteins and co-repressor complexes, and by sterically hindering the binding of transcriptional activators (Du et al, 2015; Jones, 2012; Law and Jacobsen, 2010). Together, these marks form mutually reinforcing pathways that stabilize silent chromatin domains and preserve cell identity (Du et al, 2015).

In DNA methylation, a methyl group is covalently added to the 5-carbon position of the cytosine ring (5mC), predominantly at CpG dinucleotides in mammalian genomes (Law and Jacobsen, 2010). This modification serves as a fundamental epigenetic signal that regulates genome function by recruiting methyl-binding proteins or by directly modulating DNA–protein interactions (Law and Jacobsen, 2010; Bogdanović and Veenstra, 2009; Smith and Meissner, 2013). In living cells, DNA methylation increases chromatin condensation and decreases overall DNA flexibility, even in the absence of the dedicated reader machinery, indicating that beyond serving as a docking site for chromatin factors, it can intrinsically influence the physical properties of DNA and chromatin (Buitrago et al, 2021). However, the mechanism through which DNA methylation affects the mechanics and organization of chromatin remains controversial. Computational and biophysical studies have suggested that methylation reduces DNA flexibility and disfavors nucleosome assembly, whereas other in vitro studies report enhanced histone–DNA affinity and increased nucleosome compaction (Pérez et al, 2012; Portella et al, 2013; Collings and Anderson, 2017; Choy et al, 2010; Rao et al, 2018; Ngo et al, 2016). These discrepancies likely reflect the complexity of chromatin and the context-dependent nature of nucleosome stability within higher-order structures.

Here, we address this gap by combining single-molecule force spectroscopy, fluorescence recovery after photobleaching (FRAP), and nanorheology to interrogate the effects of CpG methylation from the level of naked DNA to reconstituted nucleosomes and higher-order chromatin condensates. This integrated approach enables direct comparison of DNA methylation’s mechanical and structural impact across scales and provides a physical framework for understanding how this epigenetic modification shapes chromatin architecture and dynamics.

## Results and discussion

### CpG methylation affects the mechanical properties of DNA

**Figure 1:**
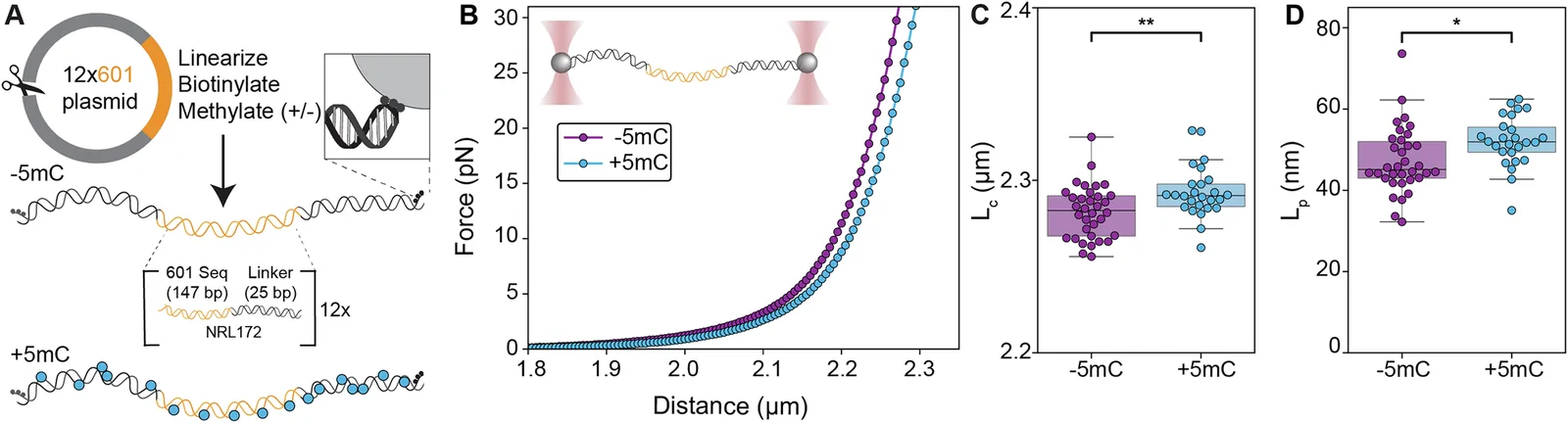
CpG methylation alters contour length (L_C_) and persistence length (L_P_) of dsDNA. (**A**) Schematic of DNA construct preparation for single-molecule force spectroscopy. A ~ 6.8 kb plasmid was linearized with the restriction enzyme NotI, and the 5’-overhangs were biotinylated using Klenow fragment. The CpG methyltransferase (MTase) M.SssI was used to methylate all cytosines at the C-5 position (5mC) within the double-stranded CG dinucleotide sequence. (**B**) Interpolation of force-extension data shows that the averaged force-distance (F,d) curves for unmethylated (−5mC) and methylated ( + 5mC) DNA follow different trajectories, reflecting variation in the mechanical properties upon CpG methylation. (**C**) CpG methylation significantly increases the DNA contour length (L_C_) (Student’s *t* test, *P* = 0.0055, *n* = 34 and *n* = 26 for -5mC and +5mC, respectively). Box plots are composed of a box from the 25th to 75th percentile with the median as a line and whiskers calculated via the Tukey method. (**D**) CpG methylation significantly increases the DNA persistence length (L_p_) from 47 ± 8 nm (unmethylated) to 52 ± 7 nm (methylated) (Student’s *t* test, *P* = 0.0157, *n* = 34 and *n* = 26 for −5mC and +5mC, respectively). Box plots are composed of a box from the 25th to 75th percentile with the median as a line and whiskers calculated via the Tukey method. Source data are available online for this figure.

**Figure EV1:**
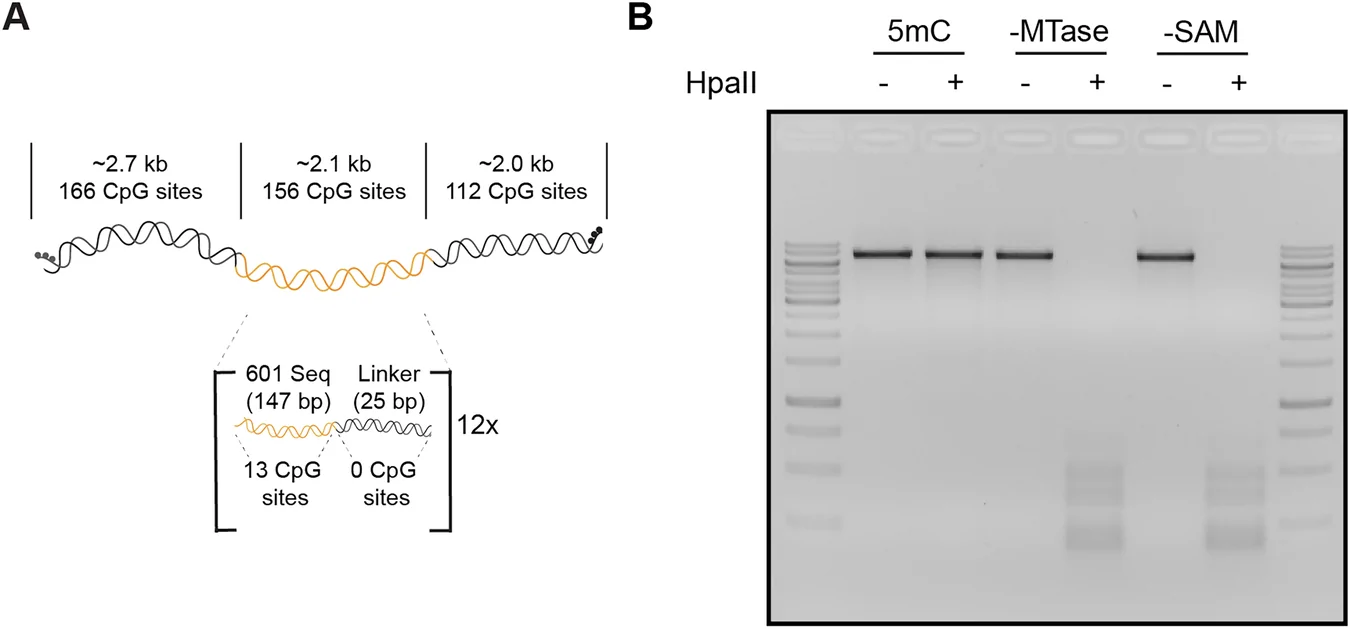
Verification of CpG methylation of the DNA construct. (**A**) The CpG methyltransferase (MTase) M.SssI was used to methylate all cytosines at CpG sites within the DNA construct. The number of CpG methylation sites present within each DNA feature, including the Widom 601-positioning sequences, linker DNA, and flanking DNA handles, is indicated. (**B**) Digestion with the methylation-sensitive restriction enzyme HpaII confirmed complete methylation: the DNA was protected from cleavage following MTase treatment (lane 3). In the absence of MTase or SAM, the DNA remained sensitive to HpaII digestion (lanes 5 and 7). The fully methylated DNA construct was used for force spectroscopy and as the template for chromatin reconstitution.

Optical tweezers enable direct mechanical characterization of DNA by tethering single DNA molecules between two optically trapped beads and applying controlled stretching forces. To investigate the effect of cytosine methylation on the mechanical properties of DNA, a ~ 6.8 kb plasmid containing 12 repeats of the Widom 601 nucleosome positioning sequence was linearized, and the 5’-overhangs were biotinylated to allow tethering to trapped beads in our optical tweezers. The CpG methyltransferase (MTase) M.SssI was used to methylate all the 434 cytosines (156 within the nucleosome positioning sequences, 13 per 601 core sequence, the remainder in the handles) at the C-5 position present within the double-stranded CG dinucleotide sequence (Figs. 1A and EV1A). Digestion with the CpG methylation-sensitive restriction enzyme HpaII confirmed that, in the presence of the MTase and methyl donor S-adenosylmethionine (SAM), the DNA was fully methylated and protected against degradation (Fig. EV1B).

Single-molecule force spectroscopy was conducted by tethering the linear biotinylated DNA construct between two streptavidin-coated polystyrene beads that were optically trapped in a dual optical trap setup. Displacement of one of the traps was performed with a constant velocity of 20 nm/s. The elastic response of the tethered DNA induces forces on the stationary bead that are registered using back focal plane detection of scattered optical trapping light. The averaged Force,distance (F,d)-curves showed qualitative differences in the trajectories between methylated ( + 5mC) – and unmethylated (−5mC) DNA (Fig. 1B).

To relate these differences in elastic response to the mechanical properties of DNA, Odijk’s extensible worm-like chain (eWLC) model was used (Odijk, 1995). Fitting the force trace data to this model revealed that CpG methylation caused a modest but significant increase in the contour length (L_*c*_) of DNA (Fig. 1C), suggesting a slight increase in the spacing between base pairs. Additionally, the persistence length (L_*p*_), which reflects DNA structural flexibility, also increased upon methylation, indicating that +5mC DNA is less flexible than -5mC DNA (Fig. 1D). These observations are consistent with previous biophysical studies reporting that methylation increases DNA contour length (Zaichuk and Marko, 2021) and stiffness (Ngo et al, 2016; Kameda et al, 2021; Kaur et al, 2012; Pérez et al, 2012; Battistini et al, 2021; Nathan and Crothers, 2002). However, previous work also reported a decreased persistence length for methylated DNA (Zaichuk and Marko, 2021), underscoring that methylation effects can be sensitive to sequence context, methylation density, and ionic conditions (Severin et al, 2011).

Together, these data suggest that CpG methylation slightly extends the DNA helix while reducing its structural flexibility. These findings support the idea that DNA methylation can directly alter the intrinsic mechanical properties of DNA independently of reader proteins, providing a physical basis for how methylated DNA might engage differently with the histone octamer.

### CpG methylation decreases the stability of the nucleosome inner turn and alters DNA wrapping

**Figure 2:**
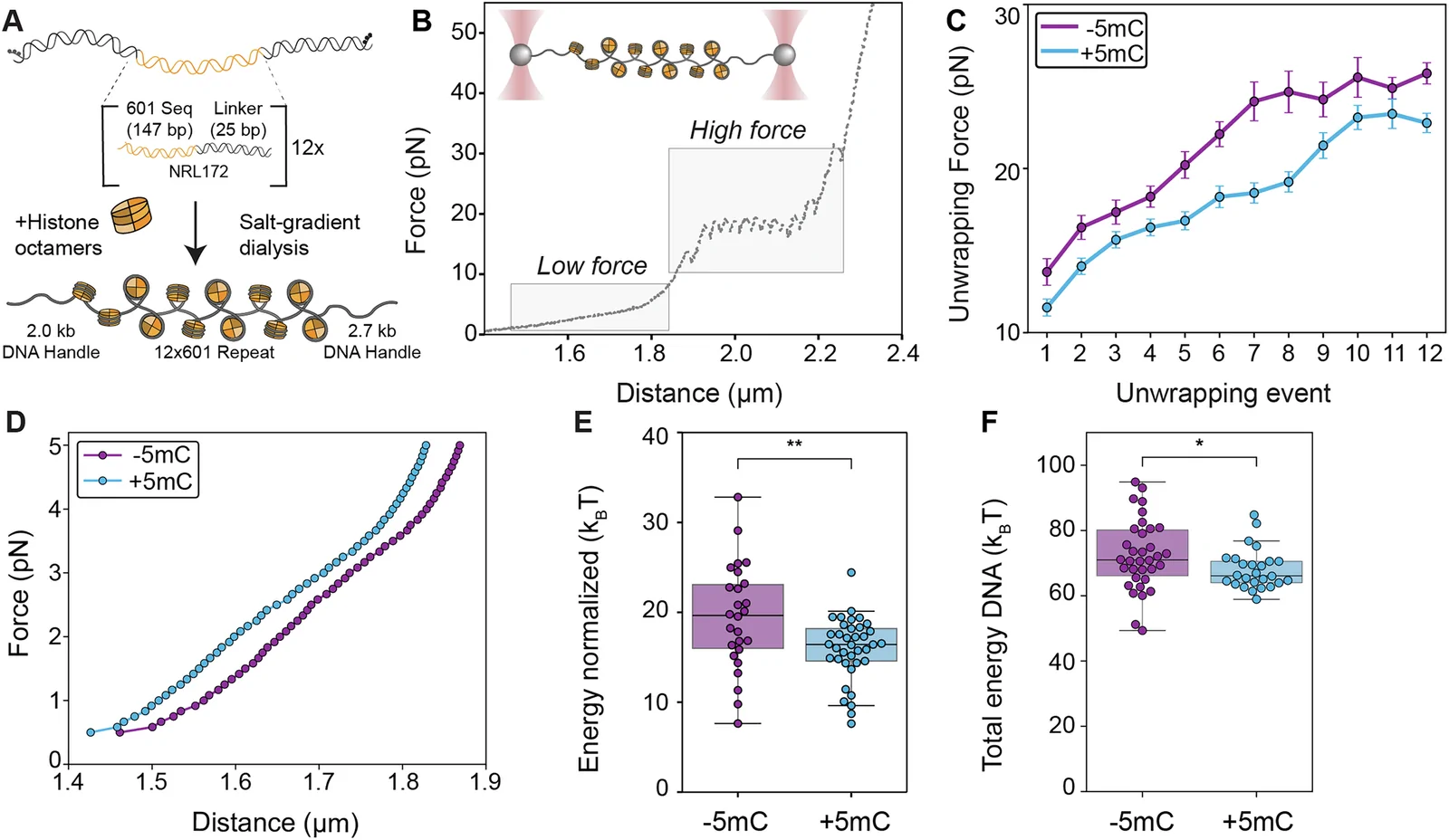
CpG methylation weakens nucleosome stability and lowers the energy required to stretch chromatin fibers. (**A**) Schematic of chromatin reconstitution. 12×601 DNA template and recombinant human histone octamers were assembled into nucleosomal arrays by salt-gradient dialysis. Gradual reduction of salt promotes spontaneous nucleosome formation at each 601-positioning sequence. (**B**) Representative F,d-curve of a 12-mer nucleosomal array. Chromatin fibers were tethered between optically trapped streptavidin-coated polystyrene beads and stretched by gradual displacement of one trap. The force response of chromatin presents two regimes: a low-force regime attributed to intra-nucleosome interaction unstacking and outer turn DNA unwrapping, and a high-force regime corresponding to unwrapping of the nucleosome inner turns. (**C**) The force required to unwrap the nucleosome inner DNA turn is significantly lower in +5mC arrays, indicating that DNA methylation destabilizes the inner turn (Mann–Whitney *U* test. *P* < 0.001 for 6–8; *P* < 0.01 for 5; *P* < 0.05 for 1,2, 4, 9; ns for 3, 10, 11, 12). Error bars represent SEM. (**D**) Force trace data were interpolated to obtain average F,d-curves for stretching −5mC and +5mC chromatin fibers in the low-force regime (0.5 pN < F < 5 pN). Similarity in the trajectories implies that unfolding of the compacted chromatin fiber is comparable for both conditions. (**E**) Normalizing total energy by the number of nucleosomes per fiber reveals a significant decrease for methylated arrays (Student’s *t* test: *P* = 0.0042, *n* = 37 and *n* = 26 for +5mC and −5mC, respectively). Box plots are composed of a box from the 25th to 75th percentile with the median as a line and whiskers calculated via the Tukey method. (**F**) The energy required to stretch the DNA in the same regime is significantly reduced for +5mC arrays (Mann–Whitney *U* test: *P* = 0.0481, *n* = 34 and *n* = 26 for +5mC and −5mC, respectively). Box plots are composed of a box from the 25th to 75th percentile with the median as a line and whiskers calculated via the Tukey method. Source data are available online for this figure.

**Figure EV2:**
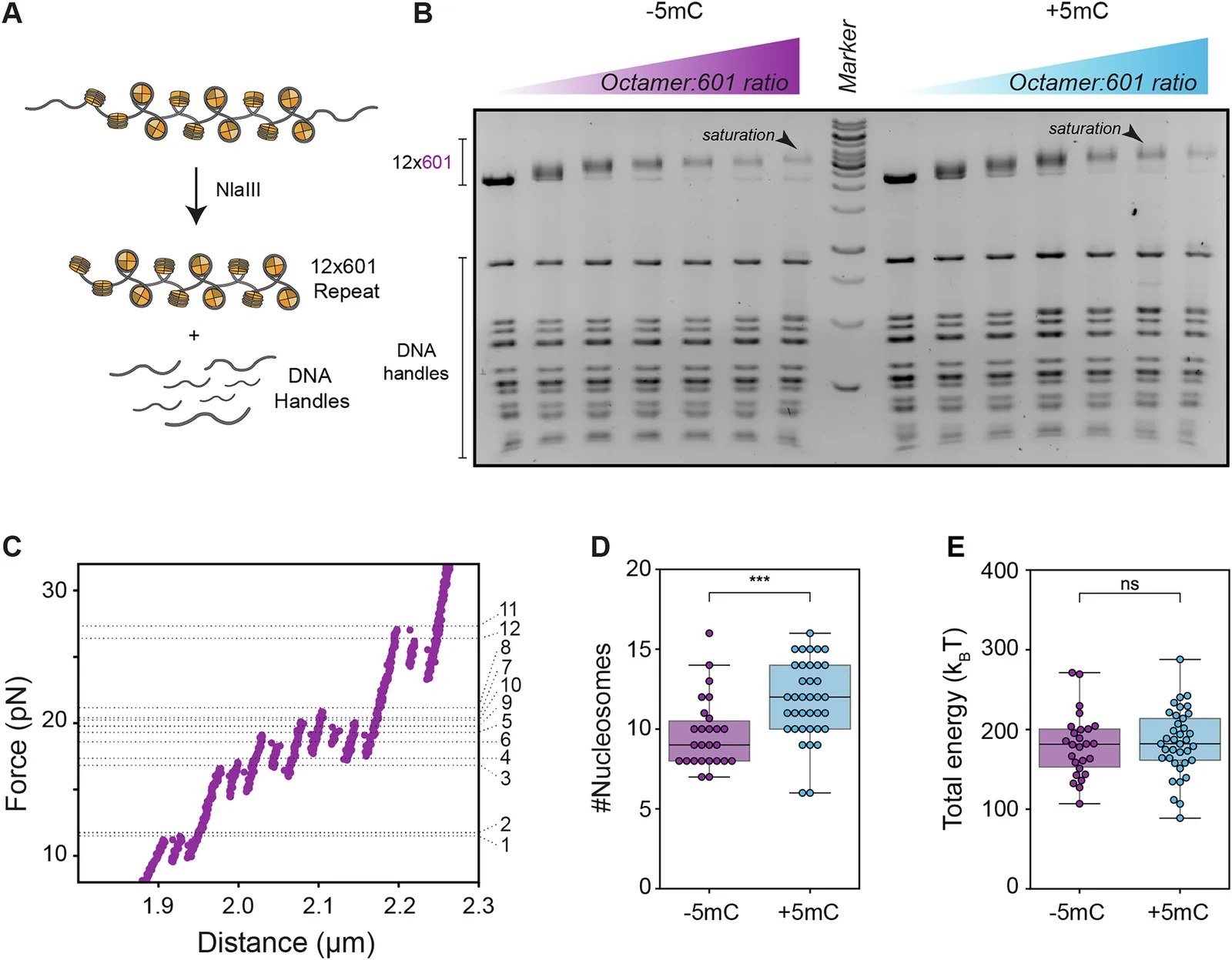
Determining the number of nucleosomes per chromatin fiber. (**A**) Enzymatic validation of nucleosome assembly. Reconstituted chromatin fibers were digested with NlaIII to distinguish between nucleosome assembly at the 601 sequences and the assembly on the DNA handles. (**B**) Saturation of nucleosome assembly determined by EMSA. Chromatin fibers were reconstituted with increasing octamer:DNA molar ratios to ensure full saturation of the 601 sites. NlaIII digestion releases the 12×601 segment (upper bands) and further digests the DNA handles (residual lower bands). Progressive mobility shifts of the 601 reflect increasing nucleosome occupancy until saturation, whereas excess octamer results in additional shifts corresponding to over-assembly on the DNA handles. (**C**) Discrete unwrapping steps in the high-force regime correspond to inner turn rupture events, enabling quantification of nucleosome number per tether. Peak forces before each rupture event were used to identify individual unwrapping transitions. (**D**) Quantification of rupture events shows a significantly higher number of nucleosomes per chromatin fiber in the +5mC condition (Mann–Whitney *U* test: *P* = 0.00039, *n* = 37 and *n* = 26 for +5mC and −5mC, respectively). Box plots are composed of a box from the 25th to 75th percentile with the median as a line and whiskers calculated via the Tukey method. (**E**) Total stretching energy (area under the curve in the low-force regime) is not significantly different between −5mC and +5mC fibers (Student’s *t* test: ns, *P* = 0.7189, *n* = 37 and *n* = 26 for +5mC and −5mC, respectively). This apparent similarity results from increased nucleosome occupancy in the 5mC condition, rather than unchanged fiber mechanics. Box plots are composed of a box from the 25th to 75th percentile with the median as a line and whiskers calculated via the Tukey method.

To determine the effect of 5mC on chromatin fibers, we reconstituted nucleosomal arrays using the same 6.8 kb DNA template as described above, containing 12 Widom 601 sequences spaced by 25 bp linkers. This results in a nucleosome repeat length (NRL) of 172 bp. Chromatin fibers were generated by salt dialysis of the template DNA with recombinant human histone octamers (Fig. 2A). Saturation of the 601 Widom sequences was confirmed by an electrophoretic mobility shift assay (EMSA) (Fig. EV2A,B).

Single-molecule force spectroscopy was used to probe the mechanical stability of reconstituted chromatin fibers, following the same approach applied to naked DNA. Different from the DNA, chromatin showed F,d-curves characterized by two distinct regimes that reflect different chromatin structural states (Fig. 2B). In the low-force regime (F < 10 pN), the applied force induces unwrapping of the outer DNA turns from nucleosomes and the disruption of any nucleosome-nucleosome interactions within the fiber(Brouwer et al, 2021). Because chromatin fibers assembled with 10n + 5 linker DNA lengths do not support intra-fiber nucleosome stacking (Gibson et al, 2019; Zhou et al, 2025), the observed response in this regime reflects nucleosome unwrapping rather than higher-order folding transitions. In the high-force regime (F > 10 pN), chromatin fibers are decompacted into a “beads-on-a-string” conformation. Further increases in the applied force led to the unwrapping of ~76 bp of inner turn DNA from each nucleosome, observed as a discrete drop in the recorded force accompanied by an increase in fiber extension. Each step corresponds to the unwrapping of an individual nucleosome, allowing estimation of nucleosome number per fiber. Consistent with a dynamic force spectroscopy model (Brower-Toland et al, 2001), the unwrapping force for both +5mC and −5mC fibers increased progressively with each event, reflecting the non-cooperative nature of inner turn unwrapping (Fig. 2C) (Pope et al, 2005). Rupture forces associated with inner turn unwrapping were quantified by identifying the peak force preceding each rupture event (Fig. EV2C), providing a measure of the resistance of DNA-histone interactions to mechanical disruption. While both +5mC and -5mC chromatin fibers show a consistent increase in unwrapping forces for each successive unwrapping event, +5mC fibers consistently showed significantly lower unwrapping forces, indicating that 5mC destabilizes the nucleosome inner turn under applied force (Fig. 2C).

Since intra-fiber nucleosome stacking interactions are negligible in NRL172 chromatin fibers, the contribution of nucleosomes to the force response of chromatin in the low-force regime is dominated by unwrapping of the nucleosomal DNA outer turns. The similar shape of the averaged F,d-curves illustrates that +5mC and -5mC chromatin fibers respond comparably under applied force (Fig. 2D). The leftward shift of the average curve for +5mC chromatin arises from the increased number of nucleosomes per fiber under this condition, which decreases the contour length L_*c*_ of the fully wrapped chromatin fiber (Fig. EV2D). Consistent with earlier experimental and theoretical studies (Nathan and Crothers, 2002; Anselmi et al, 2000, 1999), the increased persistence length of methylated DNA may reduce the entropic penalty associated with nucleosome wrapping, thereby offsetting the energetic cost of binding a stiffer polymer and potentially explaining the higher propensity for nucleosome loading.

Because stretching in the low-force regime is reversible (Brower-Toland et al, 2001; Kruithof et al, 2009), the energy required to unwrap the nucleosomal outer turn can be calculated from the total area under the curve (AUC between 0.5 and 5 pN) and normalized to the number of nucleosomes per fiber (Fig. EV2D,E). This analysis revealed that the unwrapping energy per nucleosome is lower for +5mC chromatin (Fig. 2E), indicating reduced nucleosome stability. This energy reflects contributions from both the unwrapping of the nucleosomal outer turn and the intrinsic stretching behavior of the underlying DNA. To assess the latter contribution, we quantified the energy required to stretch naked dsDNA (i.e., without nucleosomes) and found that 5mC significantly decreases the energy needed to extend DNA within this same force regime (Fig. 2F). These results indicate that both reduced nucleosome outer turn stability and altered DNA properties contribute to the mechanical behavior of methylated chromatin fibers, consistent with previous FRET experiments (Ngo et al, 2016). The observed differences in unwrapping forces between methylated and unmethylated fibers suggest that CpG methylation affects how tightly DNA is wrapped around the histone octamer.

Together, our single-molecule analyses reveal that CpG methylation modulates chromatin mechanics by altering how DNA wraps around the histone octamer, lowering the forces required for inner-turn unwrapping and reducing the energy needed to unwrap the outer turn. Thus, although methylated DNA becomes mechanically stiffer, it wraps less stably around the octamer, indicating that CpG methylation reshapes the energetic landscape of nucleosome assembly and DNA breathing. Overall, these findings show that CpG methylation reshapes chromatin properties by coupling changes in DNA mechanics to nucleosome stability and wrapping geometry.

### CpG methylation decreases chromatin dynamics within condensates

**Figure 3:**
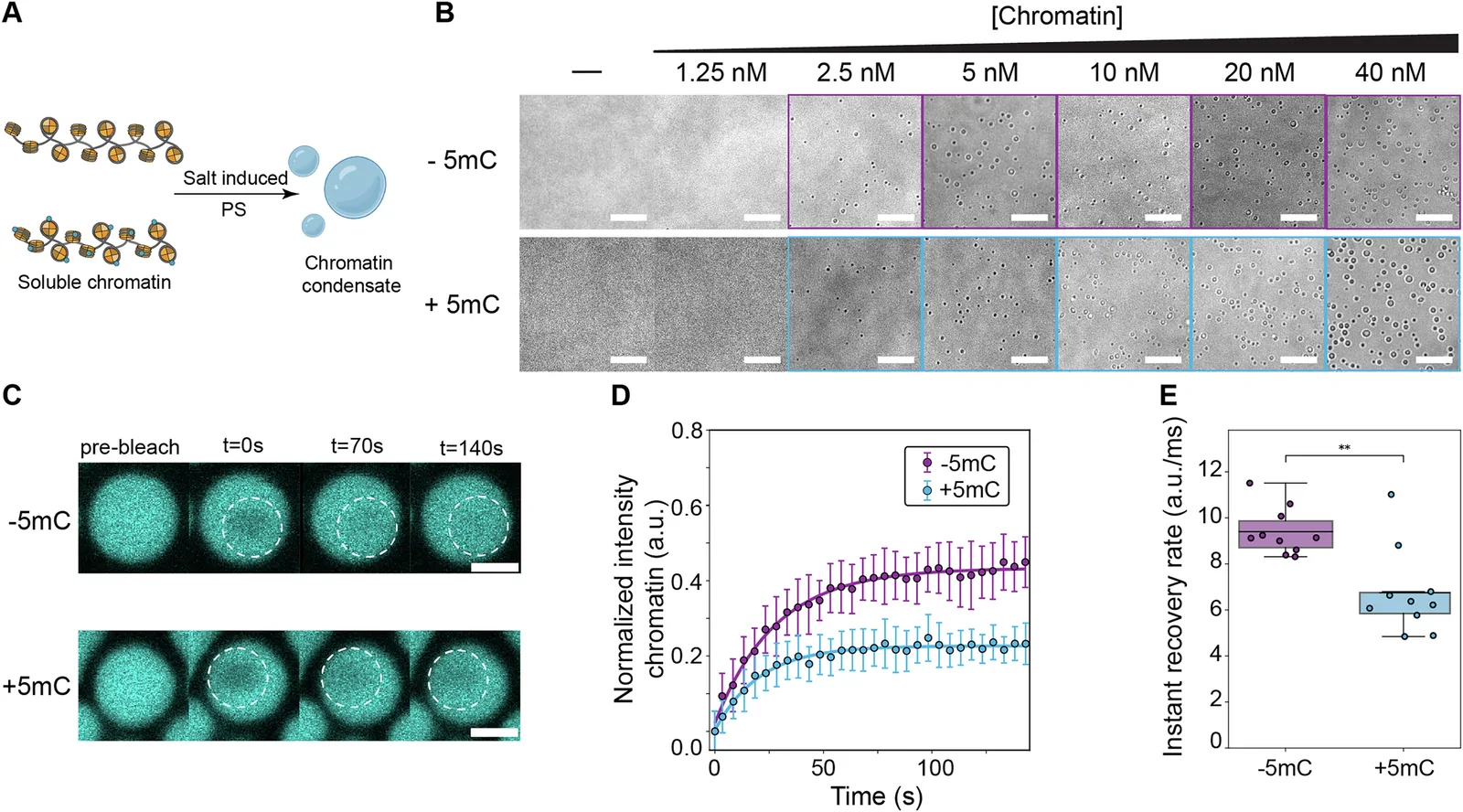
CpG methylation modulates chromatin condensate dynamics. (**A**) Schematic of the chromatin phase separation assay induced by salt. (**B**) Increasing concentrations of −5mC and +5mC chromatin fibers were incubated at 150 mM NaCl and imaged by bright-field optical microscopy. Squares highlight conditions in which condensates are observed. Scale bar = 10 µm. (**C**) Representative microscopy images of Cy5-H2B-labeled chromatin droplets before and after partial photobleaching. Scale bar = 2 µm. (**D**) Normalized fluorescence recovery curves for –5mC and +5mC condensates (*n* = 10 droplets per condition). Mean is reported, and error bars represent standard deviation. (**E**) Instantaneous recovery rates, derived from the initial slopes of the recovery curves, show slower chromatin dynamics in +5mC condensates compared to −5mC (*n* = 10 droplets per condition). Box plots are composed of a box from the 25th to 75th percentile with the median as a line and whiskers calculated via the Tukey method. Source data are available online for this figure.

**Figure EV3:**
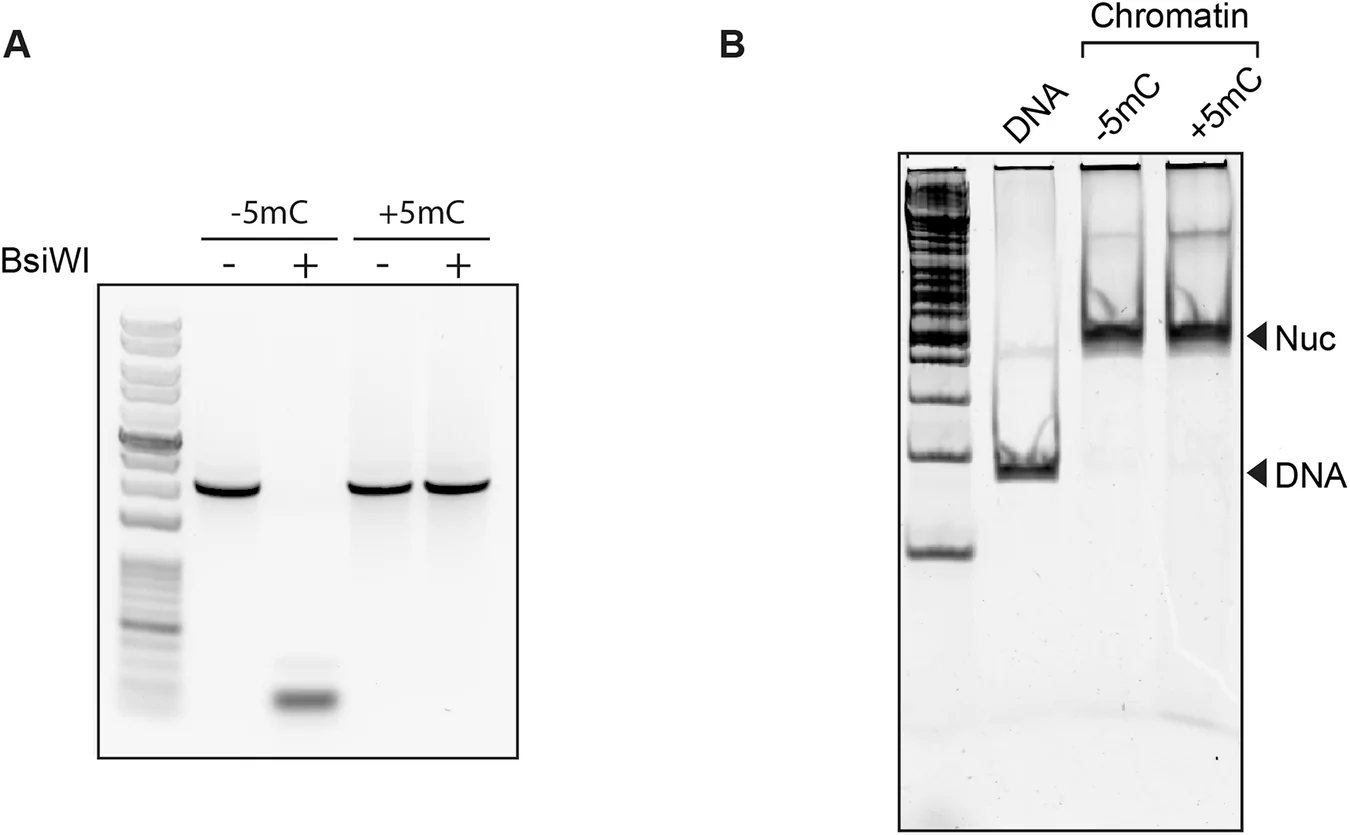
Chromatin quality control. (**A**) Digestion with the methylation-sensitive restriction enzyme BsiWI confirmed complete methylation. DNA was protected from cleavage following treatment with MTase and SAM. The methylated DNA construct was used as the template for chromatin reconstitution. (**B**) Enzymatic validation of chromatin assembly. Naked dsDNA and reconstituted chromatin fibers were digested with BstXI and analyzed on a native gel. Both −5mC and +5mC chromatin show complete and comparable nucleosome occupancy.

To determine how methylation influences higher-order chromatin organization, we next examined the phase separation behavior of methylated and unmethylated fibers. Chromatin phase separation reflects multivalent inter-nucleosome interactions that drive condensate formation, providing a direct readout of how DNA methylation affects these contacts (Sanulli et al, 2019b; Gibson et al, 2019). We used chromatin fibers containing 12 nucleosomes assembled on Widom 601-positioning sequences with 25 bp linker DNA, matching the construct used for optical tweezers measurements, but without the flanking DNA handles. Under physiological salt conditions, both –5mC and +5mC chromatin fibers underwent phase separation at comparable threshold concentrations (c_phase_ ≈ 2.5 nM), indicating that CpG methylation does not substantially alter the overall thermodynamic forces driving phase separation (Figs. 3A,B and EV3).

Given the observed differences in nucleosome wrapping and fiber architecture (Fig. 2), we next asked whether CpG methylation affects chromatin dynamics within phase-separated assemblies, even when the threshold concentration for phase separation remains unchanged. To test this, we incorporated Cy3-labeled H2B histones into chromatin fibers and performed FRAP. A defined region within each condensate was photobleached, and fluorescence recovery was monitored over time (Fig. 3C). Remarkably, both the extent (Fig. 3D) and rate (Fig. 3E) of recovery were reduced in +5mC chromatin compared to −5mC, consistent with slower diffusion and a smaller mobile fraction of chromatin in the dense phase. Importantly, these changes occur despite similar phase separation thresholds, indicating that CpG methylation does not primarily alter the overall thermodynamic propensity for condensation but instead reorganizes the interaction network and stabilizes the internal dynamics of the condensed chromatin state.

Together, these observations suggest that the local effects of CpG methylation on DNA mechanics and nucleosome stability propagate across scales to alter the material properties of chromatin condensates. While methylation increases DNA rigidity and weakens nucleosome wrapping, these changes ultimately promote a chromatin state with reduced mobility.

### CpG methylation produces denser and more viscoelastic chromatin condensates

**Figure 4:**
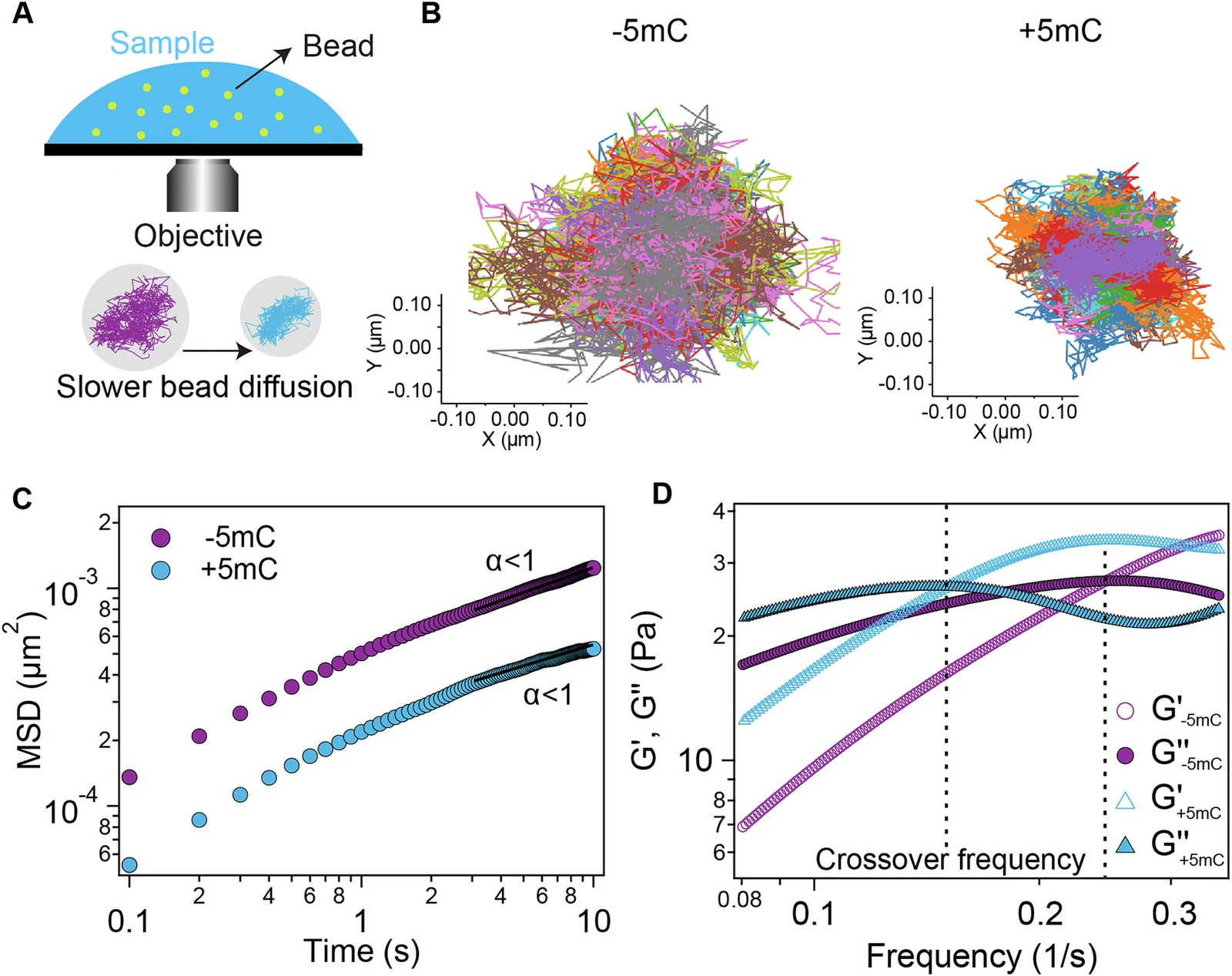
CpG methylation alters the material properties of chromatin condensates. (**A**) Schematic representation of VPT nanorheology employed to quantify the material properties of chromatin condensates. The upper panel shows beads embedded inside a condensate, and the lower panel depicts bead trajectories with altered diffusivity inside condensates with different material properties. (**B**) Trajectories of the beads tracked inside the −5mC (left) and +5mC (right) chromatin condensates. Different colors represent trajectories of distinct beads tracked inside the condensates. (**C**) Ensemble-averaged mean-squared displacement (MSD) of 200 nm carboxylate-modified beads inside −5mC and +5mC chromatin condensates. The beads exhibited subdiffusive motion inside both types of condensates, depicted by the diffusivity parameter, α<1. (**D**) The storage and loss moduli of the −5mC and +5mC chromatin condensates were estimated utilizing the Evans method(Evans et al, 2009) from the MSDs shown in (**C**). The dotted lines depict the frequency at which the crossover between storage modulus (Gʹ) and loss modulus (Gʹʹ) was observed for these condensates. Source data are available online for this figure.

The reduced mobility of +5mC chromatin within condensates suggested that CpG methylation may alter the condensates’ underlying material properties and inter-nucleosome connectivity. To directly probe their viscoelastic behavior, we performed video particle tracking (VPT) nanorheology (Alshareedah et al, 2024b, 2024b) (Fig. 4A). In this approach, the motion of 200 nm fluorescent beads, which were passively embedded into the chromatin condensates, was tracked over time. Beads were more dynamic in −5mC than in +5mC chromatin condensates, as evident from the spatial extent of the individual bead motion (Fig. 4B) and the significantly higher ensemble-average mean-squared displacement (MSD) of beads in the unmethylated condition (Fig. 4C). In both samples, the beads showed subdiffusive motion with the diffusivity parameter, α < 1, signifying the viscoelastic nature of the condensates arising from a strong intra-condensate nucleosome interaction network.

To quantify viscoelasticity, we employed the Evans method and utilized MSDs estimated from VPT measurements as input to compute the complex shear moduli (Fig. 4D) (Alshareedah et al, 2021; Evans et al, 2009). The frequency‑dependent viscoelastic moduli revealed that both types of chromatin condensates ( ± 5mC) exhibited characteristic viscoelastic behavior. Furthermore, +5mC chromatin condensates showed a crossover between the storage modulus (Gʹ) and the loss modulus (Gʹʹ) at a lower frequency compared to −5mC chromatin condensates. The inverse of this frequency, termed the crossover frequency, provides an estimate of the terminal relaxation time (τ) of the interaction network, which reflects the timescale required for the condensate network to reorganize. +5mC condensates exhibited a longer relaxation time (${\tau }_{+5{mC}}$ ~ 6.67 ± 0.56 s) compared to -5mC chromatin condensates (${\tau }_{-5{mC}}$ ~ 4.02 ± 0.95 s). This difference indicates that CpG methylation gives rise to a more viscoelastic chromatin network, in which the intra-condensate interaction network relaxes more slowly.

**Figure EV4:**
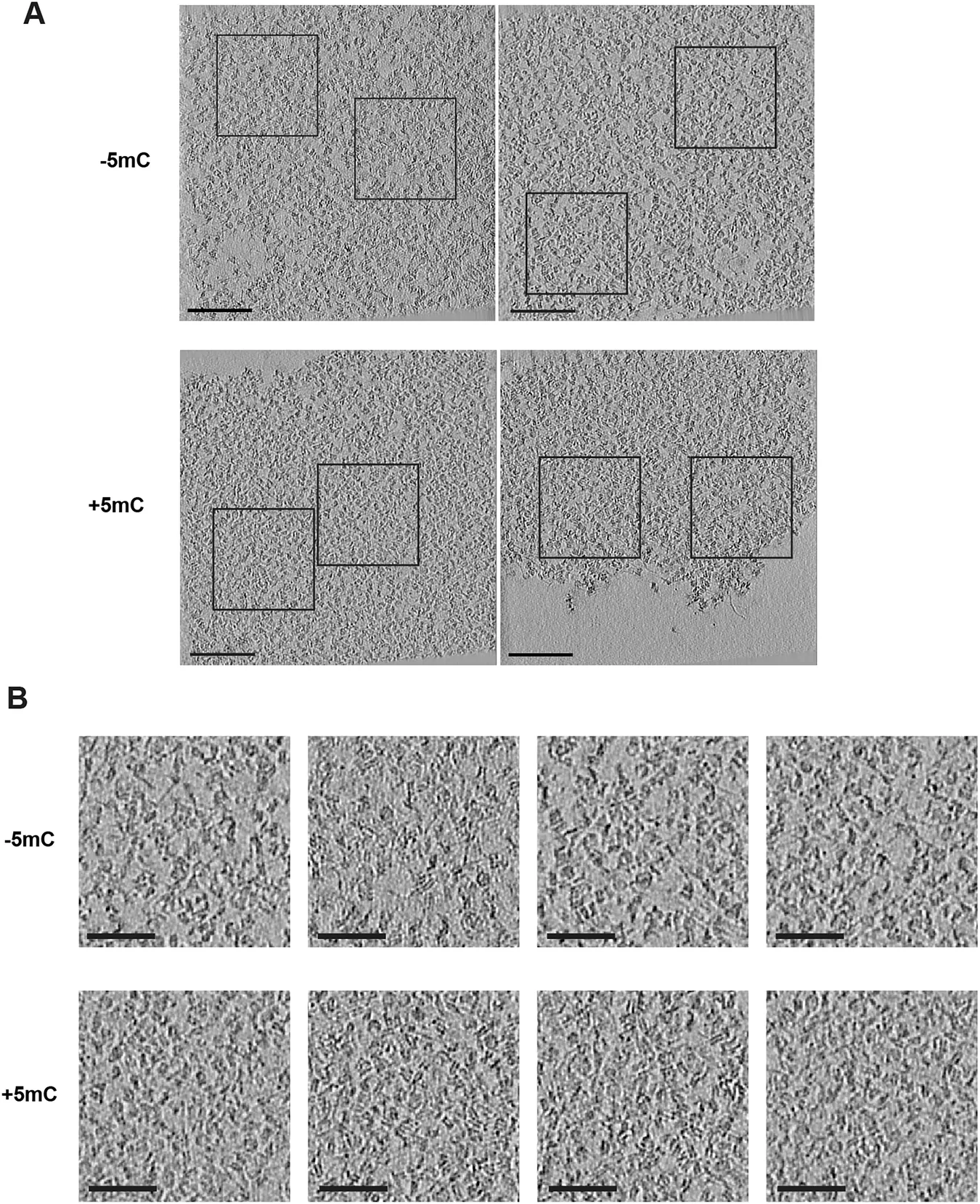
Qualitative assessment of chromatin condensates by Cryo-ET. (**A**) Representative enhanced views generated by summing five consecutive tomogram slices of −5mC (top) and +5mC (bottom) chromatin condensate. (**B**) Magnified views of the regions indicated by black squares in (**A**), highlighting differences in internal organization. Scale bar = 50 nm.

Because these mechanical differences suggested corresponding changes in condensate architecture, we next examined their nanoscale organization using cryo-electron tomography (cryo-ET). Chromatin condensates formed with 25-bp linker arrays at physiological salt were vitrified by plunge freezing, imaged with tilt series acquisition, and reconstructed into tomograms that were denoised to enhance structural features. The tomograms showed subtle qualitative differences in the internal organization of −5mC versus +5mC chromatin condensates (Fig. EV4A,B). Whereas −5mC condensates displayed a more open architecture, +5mC condensates presented a denser internal organization. These data provide qualitative structural visualization of the condensates and are consistent with the increased viscoelasticity of CpG-methylated condensates quantified by VPT-based nanorheology (Fig. 4C,D).

In summary, these observations indicate that CpG methylation remodels the material properties and nanoscale architecture of chromatin condensates without substantially altering the threshold concentration for phase separation. Rather than changing the overall thermodynamic propensity for condensation, CpG methylation reorganizes the internal interaction network of the dense phase, producing a more crosslinked chromatin network with slower internal dynamics.

Integrating these mesoscale observations with our single-molecule analyses further suggests that the local effects of CpG methylation on DNA mechanics and nucleosome dynamics propagate across scales to regulate higher-order chromatin organization. Specifically, increased DNA stiffness together with enhanced nucleosomal DNA unwrapping likely promote local rearrangements and exposure of interaction surfaces within chromatin fibers, facilitating more stable inter-nucleosome interactions and thereby strengthening the condensate network. This counterintuitive phenomenon, in which nucleosomes become locally less stable while chromatin assemblies become more viscoelastic and dynamically constrained, highlights the distinction between nucleosome-scale conformational dynamics (e.g., DNA breathing and nucleosome conformational changes) and mesoscale inter-nucleosome interactions between chromatin fibers. These findings are consistent with previous work showing that nucleosome plasticity can facilitate chromatin condensation by promoting multivalent inter-nucleosome interactions (Sanulli et al, 2019b). Thus, although CpG methylation destabilizes local nucleosome wrapping, it enhances condensate connectivity, resulting in reduced large-scale chromatin mobility and a more crosslinked chromatin network.

### CpG methylation alters chromatin organization and cooperates with H3K9me3 to promote HP1α-mediated condensation

Chromatin compaction and gene silencing within heterochromatin are orchestrated by an interplay between DNA methylation and the heterochromatin protein 1α (HP1α). HP1α recognizes the histone mark H3K9me3 through its chromodomain and oligomerizes to bridge nucleosomes, thereby promoting chromatin condensation (Canzio et al, 2011; Keenen et al, 2021; Larson et al, 2017). HP1α is a key component of repressive chromatin compartments and can itself drive chromatin phase separation in vitro (Sanulli et al, 2019b, 2019a). Notably, H3K9me3 and CpG methylation frequently co-occur within constitutive heterochromatin, where they are thought to act synergistically to stabilize compact, transcriptionally silent chromatin domains (Yang et al, 2022; Leung et al, 2014; Jabbari et al, 2019). This close spatial and functional association raises the possibility that CpG methylation influences how HP1α interacts with and organizes chromatin.

**Figure 5:**
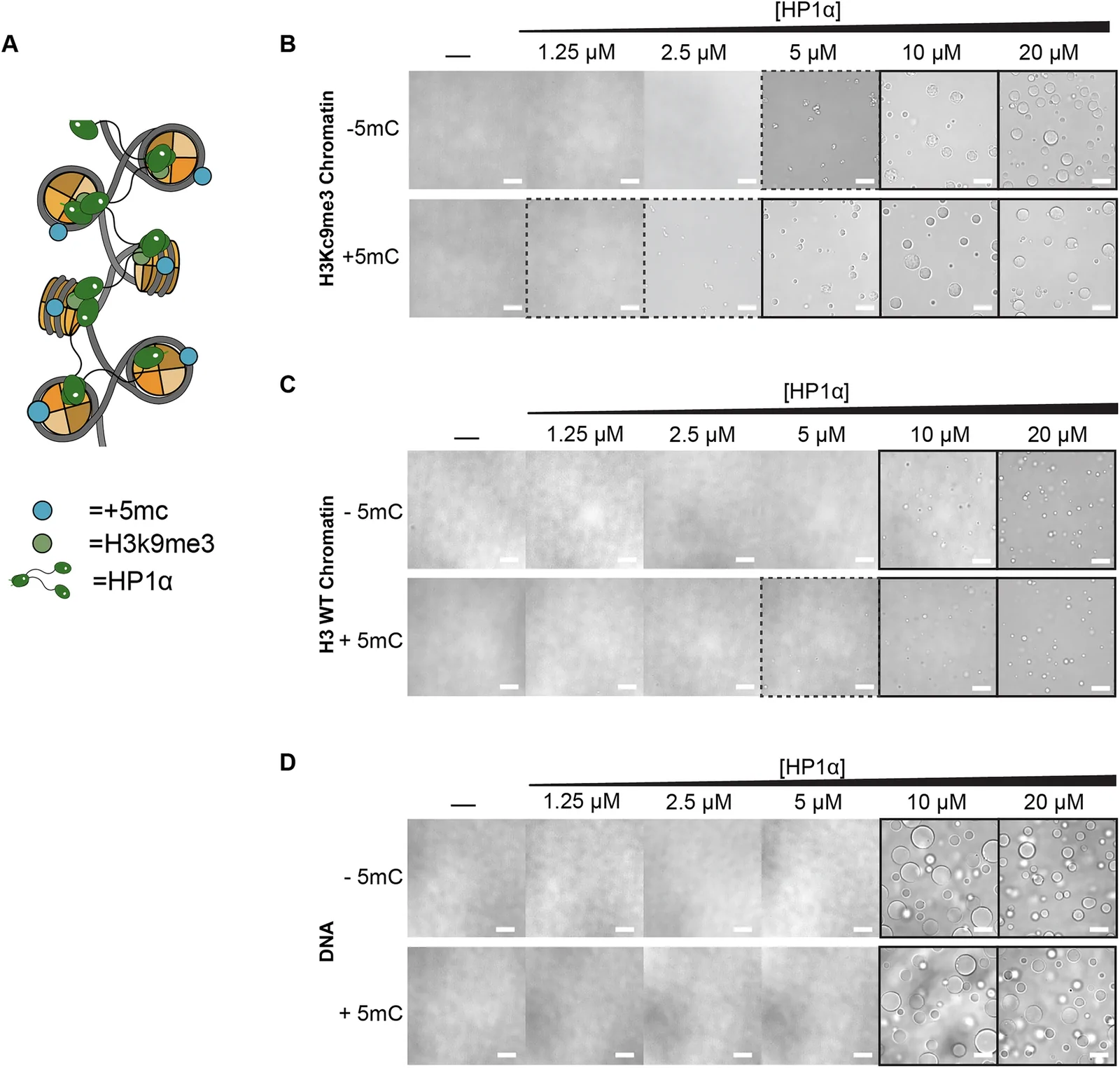
CpG methylation cooperates with H3K9me3 to promote HP1α-mediated chromatin condensation. (**A**) Schematic illustrating interactions between HP1α and chromatin fibers carrying H3Kc9me3 and CpG methylation. (**B**) Bright-field images of the indicated H3Kc9me3 chromatin condensates formed at increasing HP1α concentrations. Black squares indicate conditions in which condensates are observed. Scale bar = 4 µm. (**C**) Bright-field images of the indicated WT chromatin condensates formed at increasing HP1α concentrations. Chromatin (40 nM). Black squares mark conditions in which condensates are observed, dotted lines indicate conditions in which small irregular assemblies are observed. The complete phase diagram is shown in Fig. EV5C. Scale bar = 4 µm. (**D**) Bright-field images of condensates formed by HP1α and dsDNA (40 nM). Fixed concentrations of −5mC and +5mC dsDNA were incubated with titrated HP1α. Black squares mark conditions in which condensates are observed, showing comparable threshold concentrations for −5mC and +5mC DNA. Scale bar = 4 µm. Source data are available online for this figure.

**Figure EV5:**
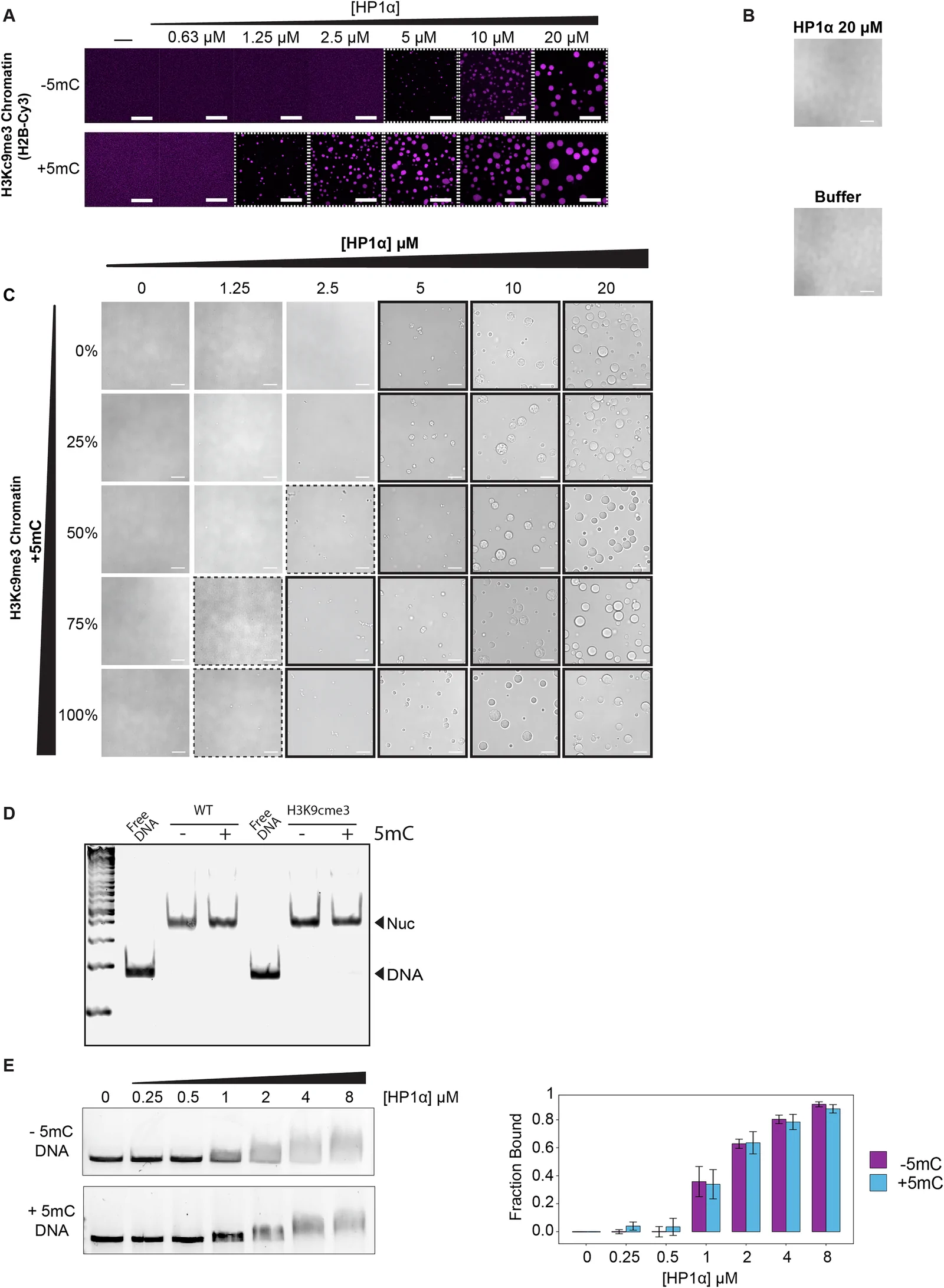
HP1α interactions with chromatin and DNA. (**A**) Confocal images of the indicated H3Kc9me3 chromatin condensates formed at increasing HP1α concentrations. Chromatin (40 nM) is fluorescently labeled. White dashed squares indicate conditions in which condensates are observed. (**B**) HP1α and buffer-only controls showing no phase separation. Scale bar = 4 µm. (**C**) Phase diagram showing HP1α-driven phase separation across varying HP1α concentrations and chromatin CpG methylation levels. Chromatin fibers contain H3Kc9me3 nucleosomes (40 nM). Black squares mark conditions in which condensates are observed, dotted lines indicate conditions in which small irregular assemblies are observed. The 0% and 100% methylation conditions correspond to those shown in Fig. 5B. Scale bar = 4 µm. (**D**) Enzymatic validation of chromatin assembly, showing complete and comparable nucleosome occupancy. Naked dsDNA and reconstituted chromatin fibers were digested with BstXI and analyzed on a native gel. (**E**) EMSA comparing HP1α binding to methylated and unmethylated dsDNA (12 × 601), showing that CpG methylation does not substantially alter HP1α–DNA interaction. Left: representative native gels; right: quantification of the fraction bound from three independent replicates. Mean ± SEM.

To test this, we reconstituted chromatin fibers containing H3K9me3 mimics generated by methyl-lysine analog chemistry (Simon and Shokat, 2012) (H3K9cme3) in the presence or absence of CpG methylation and monitored chromatin assembly formation by confocal microscopy upon addition of increasing concentration of HP1α (Fig. 5A). At a fixed chromatin concentration (40 nM), chromatin assemblies were visible at fourfold lower HP1α concentration in +5mC as compared to −5mC chromatin (5 μM versus 1.25 μM) (Figs. 5B and EV5A,B). A phase diagram experiment, in which both HP1α concentration and CpG methylation levels were titrated, further showed that the effects of CpG methylation are concentration dependent and detectable at 50% methylation (Fig. EV5C). These observations indicate that CpG methylation facilitates HP1α-mediated chromatin condensation. Interestingly, at low HP1α concentrations (1.25 and 2.5 μM), methylated chromatin formed small irregular assemblies visible in bright-field images, suggestive of early network-like assemblies prior to the appearance of larger condensates (Figs. 5B and EV5C). Consistent with the increased viscoelasticity observed for methylated chromatin (Fig. 4), these findings suggest that CpG methylation alters chromatin fiber properties and modulates the pathway of HP1α–chromatin assembly formation.

To dissect the respective contribution of H3K9me3 and CpG methylation, we next compared HP1α-driven chromatin condensation in the absence of H3K9me (WT octamers) with and without CpG methylation. As expected, HP1α exhibited a twofold higher threshold concentration for phase separation in the absence of H3K9me3 (Cc = 10 µM) (Figs. 5C,D and EV5D), consistent with its known increased affinity for this mark (Sanulli et al, 2019b; Bannister et al, 2001). In WT chromatin, CpG methylation alone produced only a modest effect, with small assemblies visible at 5 µM HP1α (Fig. 5C). Thus, while CpG methylation can promote HP1α-mediated chromatin compaction independently of H3K9me3, its effect is substantially enhanced in the presence of H3K9me3, indicating that these heterochromatin features cooperate to promote chromatin compaction.

We next asked whether this enhancement reflects increased affinity of HP1α for methylated DNA itself or instead arises from methylation-dependent changes in chromatin architecture. To distinguish between these possibilities, we assessed HP1α phase separation with naked dsDNA (40 nM, 2 kb) with or without CpG methylation. Both substrates displayed identical HP1α threshold concentration, suggesting that CpG methylation does not substantially alter the multivalent interactions between HP1α and the naked DNA required for condensate formation (Fig. 5D). We next directly tested HP1α–DNA binding using gel shift EMSA and similarly observed no difference in HP1α binding to 2 kb array DNA in the presence or absence of CpG methylation (Fig. EV5E). Thus, the increased propensity of +5mC chromatin to undergo HP1α-driven phase separation is not due to direct recognition of methylated DNA by HP1α, but rather to methylation-dependent biophysical changes within the chromatin fiber that promote more favorable HP1α engagement.

In summary, CpG methylation promotes HP1α-mediated chromatin condensation in a concentration-dependent manner and acts synergistically with H3K9 methylation to facilitate assembly formation at lower HP1α concentrations. Importantly, this enhancement is not observed with naked DNA, indicating that CpG methylation does not substantially alter direct HP1α–DNA interactions, but instead modulates higher-order chromatin interaction networks. We propose that H3K9me3 provides high-affinity binding sites for HP1α, whereas CpG methylation alters chromatin fiber organization and viscoelastic properties in a manner that increases the effective valency and stability of HP1α-mediated bridging interactions. In this way, CpG methylation cooperates with H3K9me3 to potentiate HP1α-mediated chromatin compaction by altering chromatin fiber properties rather than by directly enhancing HP1α binding to methylated DNA. More broadly, these results suggest that chromatin factors such as HP1α can sense methylation-dependent changes in chromatin mesoscale architecture even in the absence of direct recognition of the DNA modification itself. Thus, our findings support a multiscale model in which DNA methylation regulates genome organization not only by acting as a static binding platform for methyl-binding proteins but also by modulating chromatin organization across scales. More broadly, these findings illustrate how epigenetic modifications can influence genome function through both biochemical signaling pathways and emergent biophysical properties of chromatin assemblies.

## Methods

**Table:** Reagents and tools table

Reagent/resource	Reference or source	Identifier or catalog number
**Experimental models**		
**Recombinant DNA**		
pWM_12×601_25bpLinker	Addgene	#157788
**Antibodies**		
**Oligonucleotides and other sequence-based reagents**		
**Chemicals, enzymes, and other reagents**		
NotI	NEB	R0189
CpG Methyltransferase (M.SssI)		M0226M
HpaII		R0171S
NlaIII		R0125
BstXI		R0113
DrdI		R0530
BsiWI		R3553
Histone Octamers	EpiCypher	16-0001
**Software**		
Python (version 3.9.7)	https://www.python.org/	
lumicks.pylake package (version 1.2.0)	https://pypi.org/project/lumicks.pylake/	
AreTomoLive	https://github.com/czimaginginstitute/AreTomo3	
DenoiseET	https://github.com/apeck12/denoiset	
ImageJ FIJI	https://imagej.net/downloads	
IMOD	https://bio3d.colorado.edu/imod/	
**Other**		

### Cloning

Restriction-ligation cloning was used to generate a 12×601 NRL172 DNA construct for single-molecule force spectroscopy. The vector pSuperCos and the 12×601 NRL172 donor plasmid pWM_12×601_25bpLinker (Addgene, plasmid #157788) were digested with BamHI and DrdI (5 U/μg). Fragments were gel-purified (0.8% agarose in 1× TAE, 0.5 μg/ml ethidium bromide) using the NucleoSpin Gel & PCR Clean-up kit (MACHEREY-NAGEL) and ligated with T4 DNA ligase (ThermoFisher). Ligation mixtures were transformed into chemically competent DH5α, and plasmid DNA of single colonies was isolated using the Wizard® Plus SV Minipreps DNA Purification System (Promega). Plasmid sequencing was performed by Plasmidsaurus using Oxford Nanopore Technology.

### Optical tweezer construct preparation

12×601 NRL172 plasmid DNA for single-molecule experiments was amplified in methyltransferase-deficient (*dam*^*-*^*/dcm*^*-*^) chemically competent *E. coli* (NEB, C2925). Plasmid DNA was linearized with NotI (5 U/μg), yielding a construct with DNA handles of ~2.7 and 2.0 kb flanking the 601 arrays. 5’ overhangs were filled with dGTP and biotin-16-dCTP using Klenow fragment (Jena Bioscience). Biotinylated DNA was methylated using the CpG methyltransferase M.SssI (NEB). The degree of methylation was assessed by digestion using the methylation-sensitive restriction enzyme HpaII, and digestion patterns were assessed with gel electrophoresis (1.2% agarose in 1× TAE, 0.5 μg/ml ethidium bromide). The DNA construct used has a GC content of 51% and contains 482 GC dinucleotides. All DNA purifications were performed with the NucleoSpin Gel & PCR Clean-up (MACHEREY-NAGEL).

### Chromatin reconstitution

#### Eeftens lab

Chromatin reconstitution was performed by salt-gradient dialysis in 7 K MWCO Slide-A-Lyzer MINI Dialysis Devices (ThermoFisher) using a multi-headed peristaltic pump (Masterflex) based on previously established protocols (Kaczmarczyk et al, 2018). Reconstitution mixtures contained 0.179 µM 601 DNA in addition to recombinant human histone octamers (EpiCypher, 16-0001) with increasing molar ratios of histone octamer to 601 DNA. An electrophoretic mobility shift assay (EMSA) was conducted to determine the degree of saturation of nucleosome assembly at the 601 sequences. To this end, reconstituted chromatin samples were digested with NlaIII (10 U/μg) and analyzed using gel electrophoresis (0.8% agarose in 0.2× TBE, 0.5 μg/ml ethidium bromide). Nucleosomal arrays were stored at 4 °C for up to 6 weeks to ensure consistent sample quality.

#### Sanulli lab

All human histones were expressed and purified from *E. coli* following published protocols (Luger et al, 1999). Methyl-lysine analog (MLA)-containing H3 histones at position 9 (H3Kc9me3) were prepared as described previously (Simon et al, 2007). Histone labeling was performed following a previously described protocol (Yang and Narlikar, 2007). Histone octamers were assembled from purified histones by salt dialysis as described (Sanulli et al, 2019b) and purified by size exclusion using a Superdex-200 column. DNA was generated by restriction enzyme digestion of a plasmid containing 12 consecutive 601 s spaced by 25 bp (Addgene #157788), followed by size exclusion purification, as previously described (Sanulli et al, 2019b). The reconstitution of nucleosome arrays followed published protocols (Luger et al, 1999). Histone octamers are combined in equimolar amounts with 12-mer DNA (12 repeats of the 601 DNA sequence separated by 25-bp linkers). Final dialysis against Chromatin Storage Buffer (CSB: 20 mM HEPES pH 7.5, 1 mM EDTA, 1 mM DTT) was performed overnight. Assembled arrays were used for experiments within 5 days after assembly. Since the 601 repeats in the 12-mer DNA sequence are separated by BstXI restriction enzyme sites, BstXI digestion was performed, followed by native gel analysis. Over-assembly was avoided by ensuring that >95% of the digested fragments migrated as mononucleosomes rather than slower migrating species. Fluorescently labeled chromatin was prepared by incorporating 5% of octamers containing Cy3 or Cy5-labeled H2B (Yang and Narlikar, 2007).

### 12×601 DNA methylation

Purified 12×601 DNA was incubated with M.SssI CpG methyltransferase (NEB #M0226M) in NEBuffer rCutSmart (NEB) supplemented with S-adenosylmethionine (SAM #B9003S) according to the manufacturer’s recommendations. Methylation reactions were carried out at 37 °C for 4 h with gentle mixing to ensure uniform accessibility of CpG sites. Following incubation, reactions were terminated by placing them on ice and cooling to 4 °C. Successful methylation was confirmed by the expected protection of the BsiWI-sensitive site, which selectively cleaves unmethylated CpG sites. Methylated DNA was purified by ethanol precipitation and sequential 70% ethanol washes or by DNA clean-up column (Zymo Research #D4029) to remove residual methyltransferase and buffer components.

### Single-molecule force spectroscopy

Force spectroscopy experiments were performed with dual optical traps in a multi-channel laminar flow cell connected to a microfluidics system (C-Trap, LUMICKS). Prior to all measurements, the flow cell was cleaned and subsequently passivated using 20 mM HEPES pH 7.5, 150 mM NaCl, 0.5% (w/v) casein, and 0.01% (w/v) BSA. Measurements were performed in a buffer containing 20 mM HEPES pH 7.5, 100 mM NaCl, 2 mM MgCl_2_, 0.2% (w/v) BSA, and 0.02% (v/v) Tween 20. To perform force spectroscopy measurements, two streptavidin-functionalized polystyrene beads with a diameter of 1.76 µm (Spherotech BV) were optically trapped, and a force calibration was conducted. Biotinylated DNA or chromatin was flow-stretched and tethered between the trapped beads. Prior to each measurement, the force offset was set to 0 pN. Force spectroscopy measurements were performed with a constant trap velocity of 20 nm/s and forces on the stationary bead were registered using back focal plane detection of scattered optical trapping light.

### Data analysis and statistics

Data analysis was performed in Python (version 3.9.7) with custom-written scripts using the lumicks.pylake package (version 1.2.0). The analysis was not done blinded. Force trace data between 1 and 30 pN for DNA were fitted to Odijk’s extensible worm-like chain (eWLC) with force as the dependent variable to robustly solve L_*p*_ and L_*c*_ (Odijk, 1995; Broekmans et al, 2016; Wang et al, 1997). Determination of the area under the curve was achieved by numerical integration (SciPy Simpson). All F,d-curves were included to determine inner turn unwrapping forces. Unwrapping forces were plotted for all nucleosomes in the case of undersaturated or saturated fibers. The first 12 unwrapping events were included for oversaturated fibers. To obtain average F,d-curves in the low-force regime, data were interpolated and averaged. Data was tested for normality using the Shapiro–Wilk test. Normally distributed datasets were compared using Student’s *t* tests, and non-normally distributed datasets were compared with the Mann–Whitney *U* test. In all cases, the threshold of significance was set at *P* < 0.05.

### hHP1α purification

N-terminally 6X-His tagged hHP1α protein was purified from *E. coli* as previously described (Sanulli et al, 2019b). Briefly, hHP1α was affinity-purified with His-Trap followed by TEV protease treatment to cleave the N-terminal 6x-His tag. After TEV cleavage, it was subjected to a HiTrap Q HP column (GE Healthcare) and a Superdex 200HR 10/300 column (GE Healthcare). hHP1α was stored in 25 mM HEPES pH 7.5, 150 mM KCl, 1 mM DTT and 10% glycerol. Concentrations were measured by UV absorbance at 280 nm and using the calculated extinction coefficient ε = 29,495.

### Phase separation assay

Salt-induced chromatin condensates were generated by mixing a 1:1 ratio of 2× chromatin stock stored in CSB buffer and 2× phase separation buffer (300 mM NaCl, 20 mM HEPES pH 7.5, 5% glycerol), to achieve a final 150 mM NaCl. Phase separation assays with HP1α were performed by mixing 2× chromatin stock stored in CSB buffer and HP1α stored in 150 mM NaCl, 20 mM HEPES pH 7.5, 5% glycerol at a 1:1 ratio to achieve the indicated concentrations. All samples were immediately loaded onto 384-well glass-bottom plates (Greiner, #781892) precoated with PEG-silane (Laysan Bio MPEG-SIL) as previously described (Sanulli et al, 2019b; Sanulli and Narlikar, 2021) to minimize nonspecific surface interactions. Droplets were imaged using a Nikon Eclipse Ts2 microscope equipped with a 40X air objective within 1 h. Image contrast and brightness were uniformly adjusted using Fiji (ImageJ). Confocal fluorescence images were acquired on either a Zeiss LSM 880 microscope (×40 oil objective) or a Nikon Spinning Disk Confocal Microscope (×60 oil objective), omitting BSA in the glass passivation protocol to minimize droplet movement. Acquisition settings were kept constant within each experimental series. Images were processed using ImageJ FIJI.

### FRAP

FRAP experiments were performed by photobleaching Cy3-labeled proteins within condensates using 100% laser intensity at 594 nm. For chromatin condensates, H2B-Cy3 and HP1α-Cy3 were selectively bleached, and fluorescence recovery was monitored over time using time-lapse imaging at low laser power to minimize secondary bleaching. H2B-Cy3 was labeled with 50% labeling efficiency quantified spectroscopically, and the final loading of labeled biomolecules was 5%. To minimize droplet movement, BSA was omitted in the glass passivation protocol for FRAP experiments. Each recovery trace was background-subtracted and normalized to pre-bleach intensity. Experiments were performed in triplicate with at least ten droplets analyzed per condition.

### FRAP data analysis

Quantitative analysis of FRAP data was performed using custom Python scripts using data exported by the Zeiss software upon acquisition. Time–intensity traces were shifted for each droplet and aligned such that *t* = 0 corresponded to the midpoint of the bleach pulse. Each trace was background-subtracted, normalized to its pre-bleach intensity, and baseline-corrected to account for slow photobleaching during recovery. Normalized traces from at least ten droplets per condition were averaged to obtain mean recovery profiles. The early recovery phase was fit with a linear model to determine the instantaneous recovery rate, which reflects the initial diffusion-limited dynamics within condensates. Statistical comparisons between conditions (e.g., −5mC vs. +5mC) were performed using two-tailed unpaired *t* tests, with significance reported according to standard conventions (*P < 0.05, **P < 0.01, ***P < 0.001, ****P < 0.0001).

### Electron microscopy

Cryo-ET samples were prepared using Quantifoil Cu 1.2/1.3 200-mesh grids (Electron Microscopy Sciences) that were glow-discharged for 90 s at 10 mA using a PELCO easiGlow system prior to sample application. Chromatin condensate solutions (3 µL) were applied to the grids, and vitrification was performed using a Mark IV Vitrobot (Thermo Fisher Scientific) with a blot time of 1 s, blot force of 1, at 4 °C and 100% relative humidity. Grids were plunge-frozen into liquid ethane and stored in liquid nitrogen until screening and data acquisition.

### Cryo-ET data acquisition and processing

Grids were imaged with a Titan Krios G4 at 300 kV equipped with an X-FEG electron gun. Data were acquired with a Falcon 4i direct electron detector and SelectrisX energy filter set to a 10 eV slit width. Tilt series were acquired using Tomo 5 (Thermo Fisher Scientific) and a 70 µm objective lens aperture. The total dose was ~180 e/Å^2^ linearly split over 33 tilts with 3° increments and a tilt range of −48° to 48°. A pixel size of 1.2 Å/px and nominal target defocus range of 2–5 µm was used for acquisition. A total of 143 and 138 tilt series were acquired for the unmethylated and methylated conditions, respectively. Tilt series were pre-processed on-the-fly using AreTomoLive (Peck et al, 2026). This includes gain, motion, and CTF correction, followed by patch-based tilt series alignment and tomogram reconstruction with weighted back-projection. Tomogram thickness estimates were obtained from the AreTomoLive pre-processing metrics. Tomograms were denoised with DenoiseET using a pre-trained model from tomograms of synaptosomes (Cryo-ET Data Portal ID DS-10443). Search map montages and representative slices of denoised tomograms were visualized using IMOD (Kremer et al, 1996).

### Sample preparation for nanorheology

Chromatin condensates were prepared at a chromatin concentration of 400 ng/µL in the buffer containing 150 mM NaCl, 10 mM HEPES pH 7.5, 2.5% glycerol. The prepared samples, in a 10 µL volume, were incubated for 30 min in a tube and then transferred to an 18 × 18 mm microscope coverslip coated with Sigmacote (Sigma-Aldrich) solution. The coverslips were coated by immersing them in Sigmacote solution for ~2 min. After 2 min, the coverslips were subsequently dried using compressed air and then incubated at 37 °C overnight. The prepared samples were sandwiched between a 75 × 25 × 1 mm thick microscope glass slide using five layers of double-sided tape (Scotch 3 M). Further, to prevent evaporation, ~200 µL of mineral oil was injected into the chamber to surround the sample.

### VPT nanorheology

The samples for measurements were prepared as described in the previous section, with an additional step of carboxylate-modified yellow-green fluorescent 200 nm microspheres (Invitrogen) added to the sample before the induction of phase separation. The samples were then moved on to a Zeiss Primovert inverted microscope with a ×100 oil-immersion objective lens and equipped with a Teledyne FLIR Blackfly S USB3 CMOS camera. The motion of the beads inside the chromatin condensates was monitored by acquiring a 1000-frame video at a rate of 10 frames per second with a camera exposure time of 100 ms. All the measurements were repeated over three independently prepared samples.

### VPT data analysis

The VPT nanorheology framework is described in detail in our previous work (Alshareedah et al, 2024b, 2021). In brief, the TrackMate plugin of the ImageJ FIJI software was used for tracking the bead’s motion inside the condensates (Tinevez et al, 2017). The tracking provided 2D trajectories of the beads, which were further analyzed. For correcting any possible drift during the measurements, the diffusion of the center of mass of the beads was estimated from the center of mass vector ${{{\boldsymbol{R}}}}$.

1 $${{{{\boldsymbol{R}}}}}_{{{{\boldsymbol{COM}}}}}\left(k\right)={{{{\boldsymbol{R}}}}}_{{{{\bf{0}}}}}+{\sum }_{k=0}^{k}{\bar{{{{\boldsymbol{v}}}}}}_{{{{\boldsymbol{k}}}}}={{{{\boldsymbol{R}}}}}_{{{{\bf{0}}}}}+{\sum }_{k=0}^{k}\left(\frac{1}{{N}_{k}}{\sum }_{i=1}^{N}{{{{\boldsymbol{v}}}}}_{{{{\boldsymbol{i}}}},{{{\boldsymbol{k}}}}}\right)\Delta k$$

Here, ${{{{\boldsymbol{R}}}}}_{{{{\boldsymbol{0}}}}}$ is the initial center of mass vector calculated by averaging the coordinates of all beads in the first frame (k=1), ${\bar{{{{\boldsymbol{v}}}}}}_{{{{\boldsymbol{k}}}}}$ is the mean velocity of all particles in the frame k, ${{{{\boldsymbol{v}}}}}_{{{{\boldsymbol{i}}}},{{{\boldsymbol{k}}}}}$ is the velocity of the particle i in frame k in units of µm/frame and ${N}_{k}$ is the number of particles in the frame k. The quantity Δk is the frame difference, which is 1 in the present case. The drift correction was done by subtracting the center of mass from the individual bead trajectories. This was used to calculate the ensemble-averaged mean-squared displacement (MSD) using:

2 $${MSD}\left(\tau \right)={\left.\left\langle {{{\boldsymbol{R}}}}\left(t+\tau \right)-{{{\boldsymbol{R}}}}\left(t\right)\right\rangle \right|}_{t,N}$$

In Eq. (6), τ is the lag time. To understand the nature of the bead diffusion inside the chromatin condensates, we fitted the MSD to the generalized Stokes–Einstein relation,

3 $${MSD}(\tau )=4D{\tau }^{\alpha }+N$$

Here, D is the diffusion coefficient, α is the diffusivity exponent, and N is a term to account for the tracking noise.

### Estimation of dynamical moduli from nanorheology measurements

The complex and the loss moduli for the -5mC and +5mC chromatin condensates were estimated using the Evans method (Evans et al, 2009) (a custom Python script was used). The details of the complex moduli estimation from MSD are described elsewhere(Alshareedah et al, 2024a). Briefly, MSD estimated from the VPT nanorheology measurements was used to obtain the compliance j(t) using equation:

4 $$j\left(t\right)=\frac{3\pi a}{{n}_{d}{k}_{B}T} < \Delta {r}^{2}(\tau ) > $$

Here, a represents the probe particle radius (a=100 nm), ${n}_{d}$ = 2 represents the dimension of the probe particle trajectories based on which the MSDs were estimated, ${k}_{B}$ represents the Boltzmann constant, and T represents the absolute temperature at which the rheology measurements were done.

The complex shear modulus and the compliance are related through a convolution integral

5 $$\int _{0}^{\tau }G\left(t\right)j\left(\tau -t\right){dt}=\tau$$

This provides an opportunity to compute complex shear modulus by performing a Fourier transform using the relation,

6 $${G}^{*}\left(\omega \right)=\frac{1}{i \omega ^{\wedge} (\omega )}.$$

We further followed the methodology of Evans et al (Evans et al, 2009) to estimate the frequency-space complex moduli ${G}^{* }(\omega )$ using the discrete experimental data points $\left({t}_{i},{j}_{i}\right)$ using the relation,

7 $$\frac{i\omega }{{G}^{* }\left(\omega \right)}= 	\, i\omega J\left(0\right)+\frac{\left(1-{e}^{-i\omega {t}_{1}}\right)\left({J}_{1}-J\left(0\right)\right)}{{t}_{1}}+\frac{{e}^{-i\omega {t}_{N}}}{\eta }\\ 	+{\sum }_{k=2}^{N}\left(\frac{{J}_{k}-{J}_{k-1}}{{t}_{k}-{t}_{k-1}}\right)\left({e}^{-i\omega {t}_{k-1}}-{e}^{-i\omega {t}_{k}}\right)$$

Where j(0) is acquired through the extrapolation of the experimental data for compliance t=0 using a linear fit of the initial four data points and viscosity, η using a linear fit of the final ten data points following the relation, $\eta =1/{j}(t)$.

## Supplementary information

Peer Review File

Source data Fig. 1

Source data Fig. 2

Source data Fig. 3

Source data Fig. 4

Source data Fig. 5

Expanded View Figures

## Data Availability

The source data of this paper are included with the manuscript. Other data requests can be directed towards the corresponding authors. This study includes no data deposited in external repositories. The source data of this paper are collected in the following database record: biostudies:S-SCDT-10_1038-S44319-026-00894-2.
