# Supplementary material for: Multiscale physical effects of CpG methylation on DNA mechanics, nucleosome wrapping, and chromatin condensates
Source: EMBO Rep. 2026 Sep 3;27(19):5449–67. doi: 10.1038/s44319-026-00894-2 (PMC13649787; doi:10.1038/s44319-026-00894-2)
Supplement: Supplementary file 1 [file 44319_2026_894_MOESM1_ESM.pdf]

EMBO reports

Multiscale Effects of CpG Methylation on DNA Mechanics, Nucleosome Wrapping, and Chromatin Condensates

Luuk Daris, Aoon Rizvi, Ashfeen Nawar, Anurag Singh, Joshua Hutchings, Lieke Lamers, Elizabeth Montabana, Dari Kimanius, Priya R. Banerjee, Jorine Eeftens, and SERENA SANULLI

Corresponding author(s): SERENA SANULLI (ssanulli@stanford.edu) , Jorine Eeftens (jorine.eeftens@ru.nl)

Review Timeline:

Transfer Date:	24th Feb 26
Editorial Decision:	19th Mar 26
Revision Received:	5th Jun 26
Editorial Decision:	10th Jul 26
Revision Received:	16th Jul 26
Accepted:	23rd Jul 26

Editor: Esther Schnapp

Transaction Report: This manuscript was transferred to EMBO Reports following peer review at The EMBO Journal.

(Note: With the exception of the correction of typographical or spelling errors that could be a source of ambiguity, letters and reports are not edited. Depending on transfer agreements, referee reports obtained elsewhere may or may not be included in this compilation. Referee reports are anonymous unless the Referee chooses to sign their reports.)

Referee #1:

In this manuscript, the authors employed various biophysical tools to study the effect of CpG methylation on chromatin behavior.

Figure 1 examines the effect on the elasticity of naked DNA. They find that methylation makes DNA stiffer; this is in broad agreement with the literature and is therefore not novel.

Figure 2 addresses the effect on the mechanical stability of a nucleosome, specifically regarding DNA unwrapping from the histone core. They find that unwrapping is facilitated by CpG methylation. This was also previously reported in Reference 35, although the authors do not acknowledge the previous report here.

Figures 3, 4, and 5 report on the properties of condensates measured through FRAP and VPT, with and without HP1alpha. There are significant differences attributable to methylation-though not in all cases; for example, the relaxation times derived from data in Figure 4 show a minor difference that is barely larger than the error bars. However, what is unsatisfying to me, at least in the current state of the project, is that there is no mechanistic understanding of the phenomena, nor are there functional implications explored here.

Although I applaud the authors for their multipronged experimentation, and as much as I appreciate the general idea that methylation can change the properties of condensates, I am not sure EMBO J is the right venue for this work.

I also found the inclusion and discussion of their cryoET data unconvincing. They claim that there is a difference due to methylation but show only images without any quantification; consequently, it is unclear how readers are supposed to judge the validity of their claim.

Referee #2:

Referee Report for Daris et al. "Multiscale Physical Effects of CpG Methylation on DNA Mechanics, Nucleosome Wrapping, and Chromatin Condensates"
The authors investigate the role of DNA methylation of CpG dinucleotides, all the way from DNA mechanics to nucleosome stability, to chromatin condensates. A strength of the work is that it combines a range of different methods and investigates the effect of methylation at different scales and levels of molecular complexity. The authors conclude that DNA

modification can act via multiscale mechanics and not only by directing enzymes / biochemical signaling. In some places, the conclusions could perhaps be discussed more clearly and/or more critically?

Specific points of attention are outlined below.

- I am a little puzzled by the reported increase in DNA bending persistence length, in particular given that Zaichuk and Marko, using similar methods, found a clear decrease in Lp (from 47 down to 32 nm after methylation)?
- The authors report that methylation the stability of assembled nucleosomes and makes them easier to disrupt. It then seems contradictory that methylation would decrease chromatin dynamics and lead to "denser and more viscoelastic" chromatin condensates? If nucleosomes are easier to disrupt, wouldn't that lead to more dynamics and looser condensates? I am not doubting the experimental findings, I am just looking for a better intuitive explanation.
- Along the same lines, the statement "The resulting decrease in wrapping stability could facilitate additional nucleosome loading and compaction" (Line 297) seems at odds with a reduced binding energy for nucleosomes upon methylation?
- How is the "low force" regime (e.g. annotated in Figure 2B) defined?
- Minor point: Two figures are called "Figure 1".

Referee #3:

In this paper, Daris and colleagues examine how CpG methylation modulates DNA and chromatin nanomechanics, addressing a topic on which the literature has been controversial and showing effects in different directions depending on the experimental system. Here, the authors use a combination of single-molecule force spectroscopy, FRAP, and nanorheology as well as phase separation assays to determine 5mC dependent chromatin modulation. They show that 5mC increases both the contour length and persistence length of DNA, yielding longer and stiffer duplexes, while at the nucleosome level it weakens histone-DNA interactions and promotes unwrapping at lower forces. They then turn to phase separation properties and show that methylated chromatin undergoes salt-induced condensation similarly to unmethylated chromatin. However, it exhibits reduced internal mobility as measured by FRAP and increased viscoelasticity with longer relaxation times by nanorheology. Consistent with these altered material properties, CpG methylation greatly changes behavior in presence of HP1alpha, lowering the protein concentration required to drive chromatin condensation.

Overall this is an interesting study showing intriguing effects of chromatin methylation on domain formation. While there is quite some literature no effects of 5mC on DNA and chromatin mechanics, the effects on chromatin condensate formation are not well explored. Here, the authors find indeed that chromatin is less dynamic and condenses significantly easier with HP1alpha. This is very interesting to a broad readership, but I feel like this finding has to be further corroborated/more mechanistic information is needed.

1. The authors report a fourfold reduction in critical HP1alpha concentration required for chromatin condensate formation, when chromatin is methylated at CpGs. To make this more robust, the experiment should be repeated / quantified, and ideally a proper phase diagram should be generated. E.g. with one axis - degree of methylation, and the other axis - concentration of HP1alpha.
It would also be helpful if the authors provide sequence information, and localize CpGs, so the reader gets an idea how many sites are implicated.

2. Mechanistically the authors test the effect of free DNA on HP1alpha condensate formation and don't see a difference. They then state that HP1alpha - DNA interactions are not altered. I am not sure that this can be concluded. DNA binding could be directly tested using EMSA. It is also a bit strange, that HP1alpha condensation occurs around the same concentrations in the presence of naked DNA, as with chromatin containing the HP1 binding site H3K9me3. The authors do not give chromatin or DNA concentration used in these experiments either, making this difficult to interpret.

3. To further understand what is driving these differences, the authors could compare HP1 hinge mutants (which they purified according to the methods), or chromatin with and without H3K9me3. This would further allow to pinpoint the origin of the effect.

4. While the authors provide QC information on chromatin fibers for the mechanistic studies in the beginning of the paper, no information is found on chromatin assembled with H3Kc9me3 used in the HP1 assays. Generally, I am convinced that chromatin quality is critical for all these experiments, and differences in array assembly easily override effects based on DNA methylation. The data should be added.

5. The authors state that more nucleosomes are detected per fiber on 5mC chromatin for the force spectroscopy experiments. This is surprising, but could explain some of the later findings. E.g. could this increase of nucleosome loading (through whatever mechanism, maybe indicated by the somewhat stronger higher-running band in S3) also be responsible for the observed differences dynamics within droplets, i.e. dynamics and different

viscoelasticity?

6. The authors discuss their cryoET findings in a qualitative way, and provide a few images for justification, which show the claimed differences. But, of course, this is highly subjective, and possibly strongly dependent on the analyzed region, grids etc. In the absence of any quantification over a large area etc. this is really purely for illustrative purposes and no claims towards "open and porous architecture" etc. should be made.

Minor points:
7. Fig. 2C: "nucleosome number" is a bit misleading as we do not know which nucleosome is unwrapping. Rather, whichever nucleosome has the lowest stability is unwrapping first, without any spatial order. Maybe label the x-axis "unwrapping event" or something similar.

8. Fig 2C: I don't doubt the conclusions. But the statistical testing, i.e. using different tests within the same graph, seems flawed: Although the authors justify the use of whichever tests by pointwise normality testing, the t- and U-tests assess different null hypotheses. A unified statistical analysis across the whole dataset is preferable. Also, the authors show SEM, but no n is given.

Referee #1:

In this manuscript, the authors employed various biophysical tools to study the effect of CpG methylation on chromatin behavior.

Figure 1 examines the effect on the elasticity of naked DNA. They find that methylation makes DNA stiffer; this is in broad agreement with the literature and is therefore not novel.

Figure 2 addresses the effect on the mechanical stability of a nucleosome, specifically regarding DNA unwrapping from the histone core. They find that unwrapping is facilitated by CpG methylation. This was also previously reported in Reference 35, although the authors do not acknowledge the previous report here.

The reviewer is correct that previous studies have examined the effects of DNA methylation on DNA stiffness and nucleosome mechanical stability. We cite this literature in the manuscript and will ensure that ref. 35 is properly referenced in the revised version. However, an important novel contribution of our work is that we **systematically measure the effects of DNA methylation under the same experimental conditions, across three levels of chromatin organization**: naked DNA, nucleosomes within a chromatin fiber, and chromatin condensates. This unified experimental framework minimizes confounding variables such as DNA sequence, salt concentration, and experimental setup, which have varied across previous studies and likely contributed to discrepancies in the literature. In addition, it enables us to link the effects of DNA methylation on chromatin across multiple scales.

Specifically, we show that CpG methylation:

- **Increases naked DNA stiffness**. These results are consistent with several previous studies reporting methylation-induced stiffening of DNA (refs. 29, 35–39), although they differ from the findings of Zaichuk and Marko, 2021 (DOI: 10.1016/j.bpj.2021.03.039). These discrepancies further underscore the importance of performing measurements under directly comparable experimental conditions.

- **Decreases nucleosome stability within chromatin fibers**. We find that the unwrapping energy per nucleosome is lower in +5mC chromatin (Fig. 2E), indicating that DNA methylation facilitates nucleosome unwrapping. This observation is consistent with previous experimental and computational studies showing that decreased DNA flexibility promotes nucleosome unwrapping (DOI: 10.1038/ncomms10813). Importantly, our study provides additional mechanistic insights. First, our experiments measure nucleosome unwrapping within chromatin fibers, which provide a more chromatin-like context than isolated mononucleosomes examined in the previous study. Second, the previous work relied on site-specific fluorescent reporters that monitor local structural transitions at predefined sites, whereas our experiments directly measure nucleosome mechanical stability in a label-free manner through force-extension relationships, providing a complete view on the effect of 5mC on nucleosome unwrapping. Because the two approaches probe distinct physical observables, our measurements offer an independent mechanical characterization of nucleosome stability that complements previous fluorescence-based studies.

In summary, our study provides a systematic mechanical characterization of the effects of DNA methylation across chromatin scales, from free DNA to nucleosomes and chromatin fibers, within

a single experimental framework. This integrated approach allows direct comparisons across chromatin states and clarifies how DNA methylation influences chromatin mechanics across multiple levels of organization.

Figures 3, 4, and 5 report on the properties of condensates measured through FRAP and VPT, with and without HP1alpha. There are significant differences attributable to methylation-though not in all cases; for example, the relaxation times derived from data in Figure 4 show a minor difference that is barely larger than the error bars.

The relaxation times derived from the crossover frequencies in Figure 4 are $\tau \approx 6.67 \pm 0.56$ s for −5mC chromatin condensates and $\tau \approx 4.02 \pm 0.95$ s for +5mC chromatin condensates. Although the magnitude of the difference is moderate, it is statistically significant and consistent with the trends observed in the FRAP experiments. Importantly, change of this scale are meaningful for condensate network dynamics and fall within the range reported for other condensates. These results indicate that DNA methylation promotes a more constrained viscoelastic network within chromatin condensates.

However, what is unsatisfying to me, at least in the current state of the project, is that there is no mechanistic understanding of the phenomena, nor are there functional implications explored here.

We respectfully disagree that the study lacks mechanistic insight and functional implications, and we will better highlight these aspects in the revised manuscript.

At the **mechanistic level,** we find that CpG methylation alters chromatin mesoscale organization through changes in DNA physical properties and nucleosome dynamics. Specifically, the increase in DNA stiffness and DNA unwrapping from the nucleosome translates into more viscoelastic network within chromatin assemblies, indicating altered inter-nucleosome connectivity and/or increased lifetime of nucleosome-nucleosome interactions. This counterintuitive phenomenon, where **nucleosomes that are locally less stable give rise to condensates with increased viscoelasticity**, highlights the importance of distinguishing dynamics occurring within individual nucleosomes (e.g., DNA breathing and nucleosome conformational changes) from inter-nucleosome interactions that occur at the mesoscale within chromatin fibers. This concept is consistent with previous work (Sanulli et al., 2019), which showed that nucleosome dynamics facilitate chromatin condensation induced by HP1 and by salt. In that study, nucleosome conformational dynamics were proposed to expose interaction surfaces on the nucleosome core, thereby promoting multivalent inter-nucleosome interactions that drive chromatin condensation. Similarly, although individual nucleosomes become mechanically less stable upon DNA methylation, the resulting increase in conformational plasticity may allow chromatin fibers to undergo local rearrangements that facilitate network connectivity within condensate and reduce large-scale chromatin mobility. Importantly, DNA methylation does not alter phase boundaries (Fig. 3B), indicating that the thermodynamic driving forces for phase separation remain unchanged, while the internal connectivity and relaxation dynamics of the dense phase are altered.

Functionally, these findings indicate that DNA methylation influences chromatin organization by modulating inter-nucleosome interactions that are sensed by chromatin factors such as HP1. This represents a conceptual shift from the prevailing view that DNA methylation primarily regulates genome function by recruiting methyl-binding proteins or by directly modulating DNA–protein interactions. Instead, our data show that **DNA methylation alters inter-nucleosome**

interactions and thereby modulate factor binding by reshaping chromatin architecture. This implies that these mesoscale changes can influence the recruitment of factors that do not contain DNA methylation–binding domains, such as HP1.

Although I applaud the authors for their multipronged experimentation, and as much as I appreciate the general idea that methylation can change the properties of condensates, I am not sure EMBO J is the right venue for this work.

We believe this work is suited for EMBO Reports because it provides a mechanistic physical basis linking DNA methylation to heterochromatin organization. By showing that CpG methylation fundamentally alters the material properties of chromatin condensates, we provide a new layer of understanding for how this mark contributes to heterochromatin: this appears to be not just through protein recruitment, but via the intrinsic physical properties of the chromatin fiber itself.

I also found the inclusion and discussion of their cryoET data unconvincing. They claim that there is a difference due to methylation but show only images without any quantification; consequently, it is unclear how readers are supposed to judge the validity of their claim.

The cryoET data are presented in the Supplementary Information as qualitative structural observations, while the quantitative characterization of condensate properties is provided by the FRAP and VPT measurements in the main figures. Quantitative analysis of the cryoET datasets is challenging due to the high density and overlap of nucleosomes within the chromatin condensates, which currently prevents reliable particle identification and segmentation using available computational approaches. While we attempted a preliminary, coarse quantification based on global density features (Fig. R1), these analyses rely on simplifying assumptions and therefore do not provide sufficiently robust metrics to support quantitative conclusions. For this reason, and because they do not substantially add beyond the conclusions already supported by the FRAP and VPT measurements, we chose to present the cryoET data as qualitative structural observations that provide visual support for differences in chromatin organization, while relying on FRAP and VPT for rigorous quantitative assessment of condensate properties. In the revised manuscript, we will clarify this point in the text to explicitly indicate that the cryoET data are intended to provide structural context rather than quantitative analysis.

Fig. R1

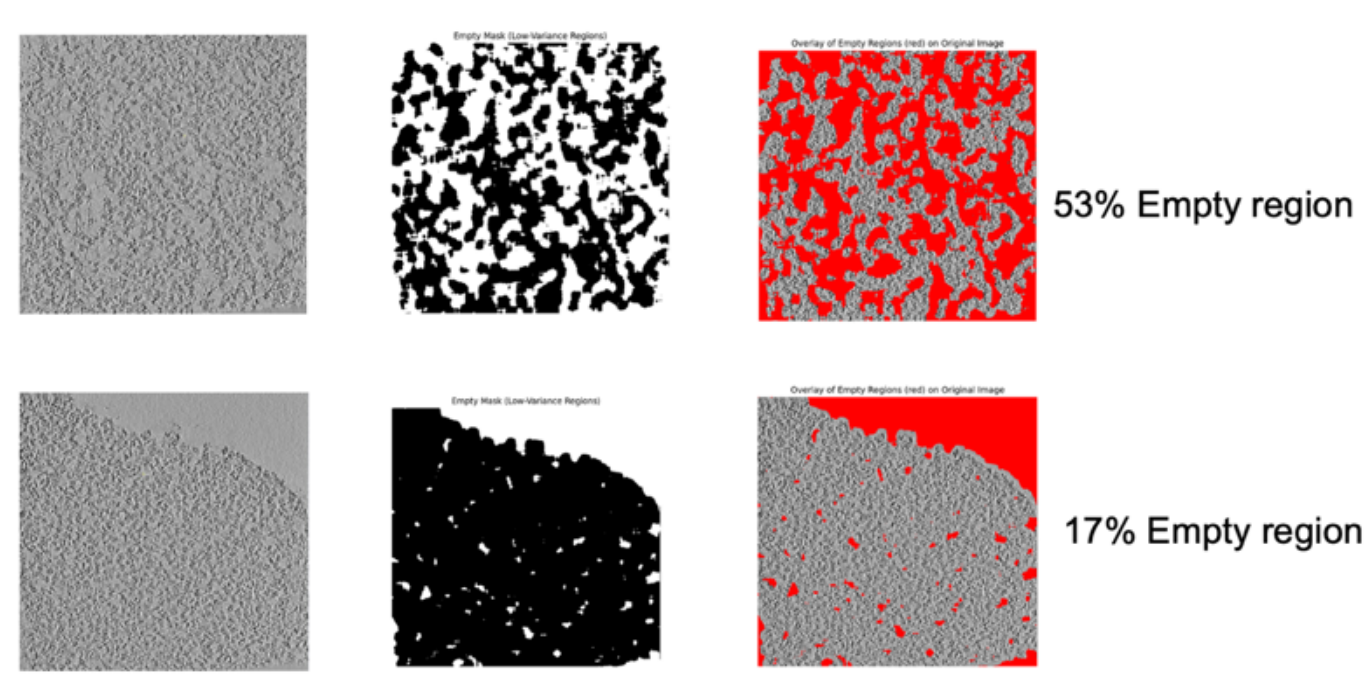

Referee #2:

Referee Report for Daris et al. "Multiscale Physical Effects of CpG Methylation on DNA Mechanics, Nucleosome Wrapping, and Chromatin Condensates"
The authors investigate the role of DNA methylation of CpG dinucleotides, all the way from DNA mechanics to nucleosome stability, to chromatin condensates. A strength of the work is that it

combines a range of different methods and investigates the effect of methylation at different scales and levels of molecular complexity. The authors conclude that DNA modification can act via multiscale mechanics and not only by directing enzymes / biochemical signaling. In some places, the conclusions could perhaps be discussed more clearly and/or more critically?

We'll expand the discussion of our conclusion and more claearly articulate the implication of these findings across multiple scales of chromatin organization.

Specific points of attention are outlined below.

- I am a little puzzled by the reported increase in DNA bending persistence length, in particular given that Zaichuk and Marko, using similar methods, found a clear decrease in Lp (from 47 down to 32 nm after methylation)?

Indeed, our results differ from the findings of Zaichuk and Marko but are consistent with several previous experimental and computational studies reporting methylation-induced stiffening of DNA (refs. 29, 35–39). Notably, these studies employed different DNA sequences and buffers, which likely contribute to some of the discrepancies between reports. The effects of CpG methylation are sequence dependent because the number and distribution of methylated sites vary between constructs, and buffer composition is also known to influence the mechanical properties of DNA (10.1016/S0006-3495(97)78780-0, 10.1002/bip.22585, 10.1529/biophysj.105.063099, DOI: 10.1126/science.1439819). Because comparing measurements across scales was important for our study, we used the same sequence for both DNA and nucleosome array experiments and performed measurements in the same physiologically relevant buffer.

- The authors report that methylation the stability of assembled nucleosomes and makes them easier to disrupt. It then seems contradictory that methylation would decrease chromatin dynamics and lead to "denser and more viscoelastic" chromatin condensates? If nucleosomes are easier to disrupt, wouldn't that lead to more dynamics and looser condensates? I am not doubting the experimental findings, I am just looking for a better intuitive explanation.

We thank the reviewer for this insightful comment. This observation, which may appear counterintuitive, is one of the most novel findings of our work, as it highlights the importance of distinguishing dynamics occurring within individual fibers from inter-nucleosome interactions that occur at the mesoscale within chromatin fibers.

Specifically, we find that increased nucleosomal DNA unwrapping driven by DNA methylation promotes nucleosome–nucleosome interactions within chromatin fibers, thereby strengthening the condensate network. This concept is consistent with previous work (Sanulli et al., 2019), which showed that nucleosome dynamics facilitate chromatin condensation induced by HP1 and by salt. In that study, nucleosome conformational dynamics were proposed to expose interaction surfaces on the nucleosome core, thereby promoting multivalent inter-nucleosome interactions that drive chromatin condensation. Although individual nucleosomes become mechanically less stable upon DNA methylation, this increased conformational plasticity allows chromatin fibers to undergo local rearrangements that facilitate intermolecular contacts while reducing large-scale chromatin mobility. In addition, partially unwrapped methylated DNA becomes more accessible for multivalent interactions. Together, these effects contribute to denser chromatin assemblies with increased viscoelasticity and internal friction within the condensate.

We will clarify and expand this point in the revised manuscript.

- Along the same lines, the statement "The resulting decrease in wrapping stability could facilitate additional nucleosome loading and compaction" (Line 297) seems at odds with a reduced binding energy for nucleosomes upon methylation?

We acknowledge that it may seem counterintuitive that the increased persistence length of methylated DNA could promote nucleosome formation. However, this effect can be explained by a reduction in the entropic penalty associated with wrapping methylated DNA, which can outweigh the energetic cost of bending a stiffer polymer. This mechanism is consistent with prior theoretical and experimental studies (doi:10.1006/jmbi.2001.5247; 10.1016/S0006-3495(00)76319-3; doi:10.1006/jmbi.1998.2575). Thus, CpG methylation can lower the energetic barrier for nucleosome formation, increasing the propensity for nucleosome loading along the DNA. Importantly, an increased propensity for nucleosome formation does not necessarily translate into greater mechanical stability of individual nucleosomes, which is consistent with our observations of reduced nucleosome unwrapping stability.

- How is the "low force" regime (e.g. annotated in Figure 2B) defined?

We thank the reviewer for this request for clarification. The definition of the ‘low force’ regime in single molecule studies varies depending on the experimental context and it typically ranges from 0 to 10 pN. In this study, we specifically define the low force regime between 0.5 and 5 pN. We will include this in the main text of the revised manuscript and cite lines here.

- Minor point: Two figures are called "Figure 1".

Referee #3:

In this paper, Daris and colleagues examine how CpG methylation modulates DNA and chromatin nanomechanics, addressing a topic on which the literature has been controversial and showing effects in different directions depending on the experimental system. Here, the authors use a combination of single-molecule force spectroscopy, FRAP, and nanorheology as well as phase separation assays to determine 5mC dependent chromatin modulation. They show that 5mC increases both the contour length and persistence length of DNA, yielding longer and stiffer duplexes, while at the nucleosome level it weakens histone-DNA interactions and promotes unwrapping at lower forces.

They then turn to phase separation properties and show that methylated chromatin undergoes salt-induced condensation similarly to unmethylated chromatin. However, it exhibits reduced internal mobility as measured by FRAP and increased viscoelasticity with longer relaxation times by nanorheology. Consistent with these altered material properties, CpG methylation greatly changes behavior in presence of HP1alpha, lowering the protein concentration required to drive chromatin condensation.

Overall this is an interesting study showing intriguing effects of chromatin methylation on domain formation. While there is quite some literature no effects of 5mC on DNA and chromatin mechanics, the effects on chromatin condensate formation are not well explored. Here, the authors find indeed that chromatin is less dynamic and condenses significantly easier with HP1alpha. This is very interesting to a broad readership, but I feel like this finding has to be further corroborated/more mechanistic information is needed.

We thank the reviewer for acknowledging the potential interest of our work to a broad readership.

1. The authors report a fourfold reduction in critical HP1alpha concentration required for chromatin condensate formation, when chromatin is methylated at CpGs. To make this more robust, the experiment should be repeated / quantified, and ideally a proper phase diagram should be generated. E.g. with one axis - degree of methylation, and the other axis - concentration of HP1alpha.

The experiments shown are representative of at least three independent replicates, and the critical concentration (Cc) is used to quantify the HP1α concentration at which condensates first appear by microscopy. These measurements consistently show an approximately fourfold reduction in the HP1α concentration required for condensate formation in the presence of CpG methylation.

To further strengthen this point, we will perform additional experiments to generate a phase diagram by titrating both the degree of DNA methylation and HP1α concentration using H3K9me chromatin. This analysis will allow a more systematic assessment of how DNA methylation shifts the phase boundary for HP1α-driven chromatin condensation.

It would also be helpful if the authors provide sequence information, and localize CpGs, so the reader gets an idea how many sites are implicated.

The 601 repeats with 25 bp linkers contain 13 methylation sites each, all of which are present within the 147 bp 601 core sequence itself. The DNA construct used for force spectroscopy experiments contains 434 CpG sites in total. A more detailed description of the CpG content and its distribution within the sequence will be included in the revised manuscript and shown in Supplementary Fig.

2. Mechanistically the authors test the effect of free DNA on HP1alpha condensate formation and don't see a difference. They then state that HP1alpha - DNA interactions are not altered. I am not sure that this can be concluded. DNA binding could be directly tested using EMSA. It is also a bit strange, that HP1alpha condensation occurs around the same concentrations in the presence of naked DNA, as with chromatin containing the HP1 binding site H3K9me3. The authors do not give chromatin or DNA concentration used in these experiments either, making this difficult to interpret.

Indeed, HP1α condensate formation is dependent both on HP1–DNA interactions and on HP1 multivalency (oligomerization) promoted by DNA binding. In our experiments, we tested the effect of free DNA on HP1α condensate formation and did not observe a difference between unmethylated and methylated DNA conditions. However, we agree with the reviewer that this alone does not allow us to conclusively determine whether HP1–DNA binding affinity is altered.

To further clarify this point, we will perform EMSA experiments comparing HP1 binding to unmethylated and methylated DNA to directly assess whether DNA methylation affects HP1–DNA interactions.

The concentrations used in these experiments are 20 nM chromatin and 40 nM DNA, which will be explicitly stated in the revised manuscript.

3. To further understand what is driving these differences, the authors could compare HP1 hinge mutants (which they purified according to the methods), or chromatin with and without H3K9me3. This would further allow to pinpoint the origin of the effect.

HP1 hinge mutants do not form condensates with chromatin, as shown in the figure R2 and in our previous work (Wong et al. 2025 doi: https://doi.org/10.1101/2025.09.21.676939), and therefore would not allow further mechanistic insight into the origin of the observed effects.

Figure for referee with unpublished data has been removed upon request by the authors.

By contrast, experiments comparing chromatin with and without H3K9me3 will help clarify the contribution of the H3K9–HP1 interaction. If H3K9me3 enables a specific HP1 binding mode distinct from that observed with unmodified chromatin, one might expect that DNA methylation would alter the critical concentration (Cc) for condensate formation only in the presence of H3K9me3 chromatin, but not with WT chromatin. Such a result would indicate that H3K9me3-dependent HP1 binding is required for sensing the effects of DNA methylation.

4. While the authors provide QC information on chromatin fibers for the mechanistic studies in the beginning of the paper, no information is found on chromatin assembled with H3Kc9me3 used in the HP1 assays. Generally, I am convinced that chromatin quality is critical for all these experiments, and differences in array assembly easily override effects based on DNA methylation.

Chromatin QC will be provided for each panel.

5. The authors state that more nucleosomes are detected per fiber on 5mC chromatin for the force spectroscopy experiments. This is surprising, but could explain some of the later findings. E.g. could this increase of nucleosome loading (through whatever mechanism, maybe indicated by the somewhat stronger higher-running band in S3) also be responsible for the observed differences dynamics within droplets, i.e. dynamics and different viscoelasticity?

The increased nucleosome loading observed in the force spectroscopy experiments arises from the different DNA construct used in those assays. Specifically, these experiments employ longer DNA handles (2.0 and 2.7 kb) lacking 601 nucleosome positioning sequences, which can allow additional nucleosome assembly along the DNA.

In contrast, the DNA construct used for the condensate experiments contains only defined 601 positioning sequences spaced by 25 bp, which prevents over-assembly and regularly position nucleosomes at the 601 sequences. Under the optimized assembly conditions used here, native PAGE analysis shows that this design yields comparable methylated and unmethylated chromatin fibers, indicating that the observed differences in condensate dynamics cannot be attributed to

increased nucleosome loading or oversaturation. We will clarify these distinctions between constructs and experimental conditions in the revised manuscript.

6. The authors discuss their cryoET findings in a qualitative way, and provide a few images for justification, which show the claimed differences. But, of course, this is highly subjective, and possibly strongly dependent on the analyzed region, grids etc. In the absence of any quantification over a large area etc. this is really purely for illustrative purposes and no claims towards "open and porous architecture" etc. should be made.

We agree with the reviewer that the cryoET images were included primarily for illustrative purposes. Quantitative analysis of these datasets is challenging due to the high density of nucleosomes within the condensates, which currently prevents reliable segmentation and particle identification across large regions.

Examples of preliminary masking-based analyses are provided below; however, these rely on simplifying assumptions and do not provide sufficiently rigorous metrics. For this reason, and because they do not substantially add beyond the conclusions already supported by the FRAP and VPT measurements, we prefer to keep the cryoET data as qualitative observations in the Supplementary Information.

Accordingly, we will tone down the language in the manuscript and avoid strong claims regarding an "open" or "porous" architecture. We will clarify that the cryoET images are intended to provide qualitative structural context that complements the quantitative measurements obtained from FRAP and VPT.

Fig. R1

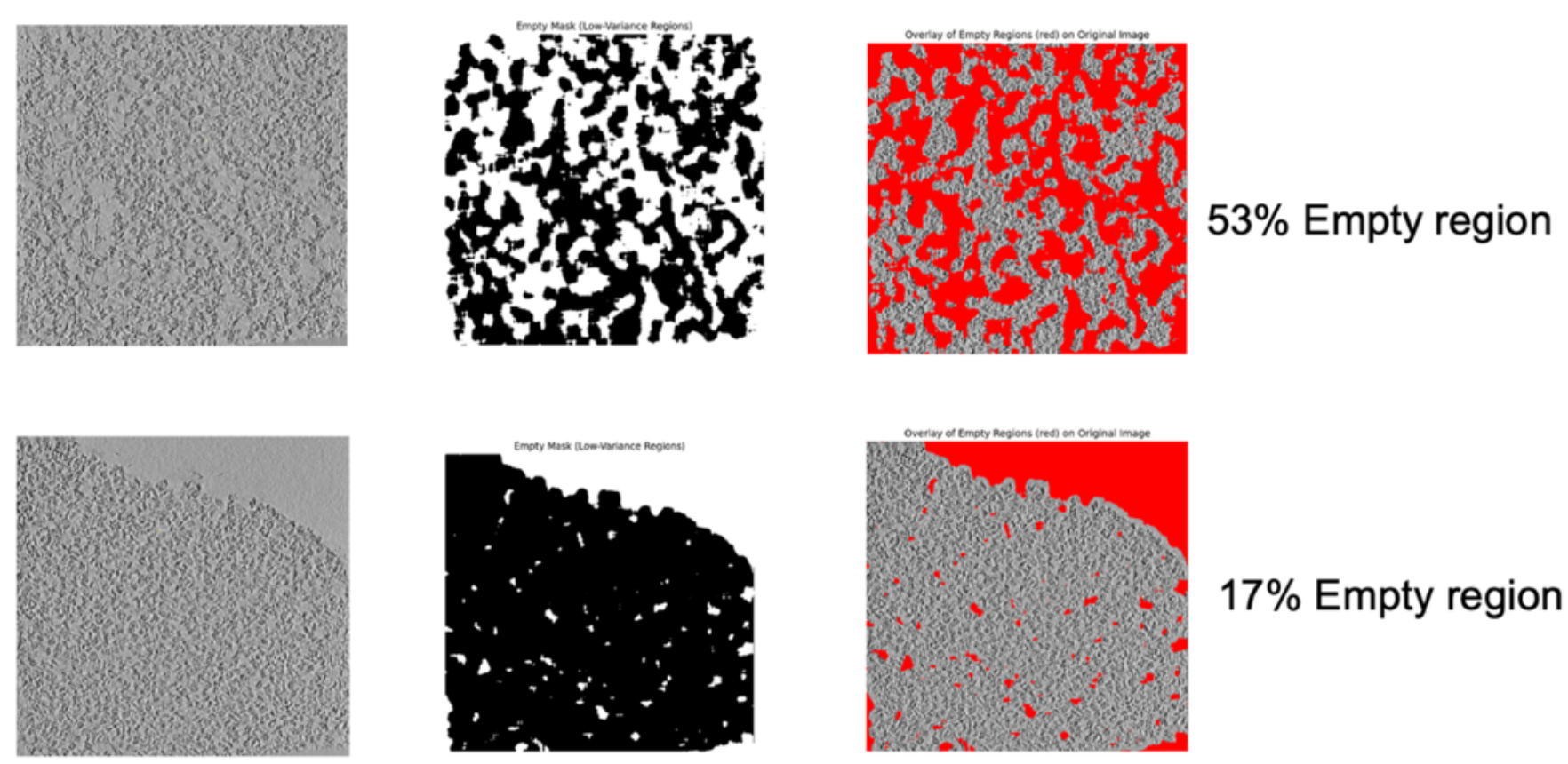

Minor points:

7. Fig. 2C: "nucleosome number" is a bit misleading as we do not know which nucleosome is unwrapping. Rather, whichever nucleosome has the lowest stability is unwrapping first, without any spatial order. Maybe label the x-axis "unwrapping event" or something similar.

We thank the reviewer for pointing this out and we will change this in the revised manuscript to read 'remaining nucleosomes'.

8. Fig 2C: I don't doubt the conclusions. But the statistical testing, i.e. using different tests within the same graph, seems flawed: Although the authors justify the use of whichever tests by

pointwise normality testing, the t- and U-tests assess different null hypotheses. A unified statistical analysis across the whole dataset is preferable. Also, the authors show SEM, but no n is given.

We thank the reviewer for pointing out the missing sample sizes and will update this in the revised manuscript. In addition, we will look for a more appropriate statistical test and we are open to suggestions from the reviewer.

1st Editorial Decision **19th Mar 2026**

Dear Serena,

Thank you for the transfer of your manuscript to EMBO reports, and for your revision plan.

I agree with your suggestions for how to address the referee concerns and would thus like to invite you to revise your manuscript with the understanding that the referee concerns must be fully addressed and their suggestions taken on board. Please address all referee concerns in a complete point-by-point response.

Acceptance of the manuscript will depend on a positive outcome of a second round of review. It is EMBO reports policy to allow a single round of major revision only and acceptance or rejection of the manuscript will therefore depend on the completeness of your responses included in the next, final version of the manuscript.

We realize that it is difficult to revise to a specific deadline. In the interest of protecting the conceptual advance provided by the work, we recommend a revision within 3 months (19th Jun 2026). Please discuss the revision progress ahead of this time with the editor if you require more time to complete the revisions.

You can either publish the study as a short report or as a full article. For short reports, the revised manuscript should not exceed 27,000 characters (including spaces but excluding materials & methods and references) and 5 main plus 5 expanded view figures. The results and discussion sections must further be combined, which will help to shorten the manuscript text by eliminating some redundancy that is inevitable when discussing the same experiments twice. For a normal article there are no length limitations, but it should have more than 5 main figures and the results and discussion sections must be separate. In both cases, the entire materials and methods must be included in the main manuscript file.

IMPORTANT NOTE: we perform an initial quality control of all revised manuscripts before re-review. Your manuscript will FAIL this control and the handling will be DELAYED if the following APPLIES:
1) A data availability section providing access to data deposited in public databases is missing. If you have not deposited any data, please add a sentence to the data availability section that explains that.
2) Your manuscript contains statistics and error bars based on n=2. Please use scatter blots in these cases. No statistics should be calculated if n=2.

When submitting your revised manuscript, please review the submission guidelines (https://link.springer.com/journal/44319/submission-guidelines#cms-Revised-submissions) and carefully review the instructions that follow below. Failure to include requested items will delay the evaluation of your revision.

1) a .docx formatted version of the manuscript text (including legends for main figures, EV figures and tables). Please make sure that the changes are highlighted to be clearly visible.

2) individual production quality figure files as .eps, .tif, .jpg (one file per figure). See hhttps://media.springernature.com/original/springer-cms/rest/v1/content/27825798/data/v1 for more info on how to prepare your figures.

3) We replaced Supplementary Information with Expanded View (EV) Figures and Tables that are collapsible/expandable online. A maximum of 5 EV Figures can be typeset. EV Figures should be cited as 'Figure EV1, Figure EV2" etc... in the text and their respective legends should be included in the main text after the legends of regular figures.

- For the figures that you do NOT wish to display as Expanded View figures, they should be bundled together with their legends in a single PDF file called *Appendix*, which should start with a short Table of Content. Appendix figures should be referred to in the main text as: "Appendix Figure S1, Appendix Figure S2" etc. See 'Expanded View' section of the submission guidelines.

- Additional Tables/Datasets should be labeled and referred to as Table EV1, Dataset EV1, etc. Legends have to be provided in a separate tab in case of .xls files. Alternatively, the legend can be supplied as a separate text file (README) and zipped together with the Table/Dataset file.

4) a .docx formatted letter INCLUDING the reviewers' reports and your detailed point-by-point responses to their comments. As part of the EMBO Press transparent editorial process, the point-by-point response is part of the Review Process File (RPF), which will be published alongside your paper.

5) a complete author checklist, which you can download from our author guidelines. Please insert information in the checklist that is also reflected in the manuscript. The completed author checklist will also be part of the RPF.

6) Please note that all corresponding authors are required to supply an ORCID ID for their name upon submission of a revised manuscript (<https://orcid.org/>), which can be linked to your profile in the manuscript submission system.

7) Before submitting your revision, primary datasets produced in this study need to be deposited in an appropriate public database. Please remember to provide a reviewer password if the datasets are not yet public. The accession numbers and database should be listed in a formal "Data Availability" section placed after Methods. Please note that the Data Availability Section is restricted to new primary data that are part of this study. * Note - All links should resolve to a page where the data can be accessed. *

If your study has not produced novel datasets, please mention this fact in the Data Availability Section.

8) At EMBO Press we ask authors to provide source data for the main manuscript figures. You will receive a separate email with instructions for providing source data with your revised manuscript, including how to upload and organize the files.

9) Our journal also encourages inclusion of *data citations in the reference list* to directly cite datasets that were re-used and obtained from public databases. Data citations in the article text are distinct from normal bibliographical citations and should directly link to the database records from which the data can be accessed. In the main text, data citations are formatted as follows: "Data ref: Smith et al, 2001" or "Data ref: NCBI Sequence Read Archive PRJNA342805, 2017". In the Reference list, data citations must be labeled with "[DATASET]". A data reference must provide the database name, accession number/identifiers and a resolvable link to the landing page from which the data can be accessed at the end of the reference.

10) Regarding data quantification, the following points must be specified in each figure legend:

- the name of the statistical test used to generate error bars and P values,

- the number (n) of independent experiments (please specify technical or biological replicates) underlying each data point,

- the nature of the bars and error bars (s.d., s.e.m.),

- If the data are obtained from n Program fragment delivered error ``Can't locate object method "less" via package "than" (perhaps you forgot to load "than"?) at //ejpvfs23/sites23b/embor_www/letters/embor_decision_revise_and_review.txt line 54.' 2, use scatter blots showing the individual data points.

Discussion of statistical methodology can be reported in the materials and methods section, but figure legends should contain a basic description of n, P and the test applied.

- Please also include scale bars in all microscopy images.

11) The journal requires a statement specifying whether or not authors have competing interests (defined as all potential or actual interests that could be perceived to influence the presentation or interpretation of an article). In case of competing interests, this must be specified in your disclosure statement.

12) All Materials and Methods need to be described in the main text using our 'Structured Methods' format, which is required for all research articles. According to this format, the Methods section includes a separate Reagents and Tools Table file (listing key reagents, experimental models, software and relevant equipment and including their sources and relevant identifiers) and a Methods and Protocols section describing the methods using a step-by-step protocol format. The aim is to facilitate adoption of the methodologies across labs. More information on how to adhere to this format as well as a downloadable template (.docx) for the Reagents and Tools Table can be found in our author guidelines.

We would also welcome the submission of cover suggestions, or motifs to be used by our Graphics Illustrator in designing a cover.

As part of the EMBO publication's Transparent Editorial Process, EMBO reports publishes online a Review Process File (RPF) to accompany accepted manuscripts. This File will be published in conjunction with your paper and will include the referee reports, your point-by-point response and all pertinent correspondence relating to the manuscript.

You are able to opt out of this by letting the editorial office know (emboreports@embo.org). If you do opt out, the Review Process File link will point to the following statement: "No Review Process File is available with this article, as the authors have chosen not to make the review process public in this case."

I look forward to seeing a revised form of your manuscript when it is ready.

Best wishes,
Esther

Esther Schnapp, PhD
Senior Editor
EMBO reports

Referee #1:

In this manuscript, the authors employed various biophysical tools to study the effect of CpG methylation on chromatin behavior.

Figure 1 examines the effect on the elasticity of naked DNA. They find that methylation makes DNA stiffer; this is in broad agreement with the literature and is therefore not novel.

Figure 2 addresses the effect on the mechanical stability of a nucleosome, specifically regarding DNA unwrapping from the histone core. They find that unwrapping is facilitated by CpG methylation. This was also previously reported in Reference 35, although the authors do not acknowledge the previous report here.

The reviewer is correct that previous studies have examined the effects of DNA methylation on DNA stiffness and nucleosome mechanical stability. We cite this literature in the manuscript and will ensure that this reference (now Ref. 34) and other relevant studies (refs. 29-33, 37-40) are referenced in the revised version. However, an important novel contribution of our work is that we **systematically measure the effects of DNA methylation under the same experimental conditions, across three levels of chromatin organization**: naked DNA, nucleosomes within a chromatin fiber, and chromatin condensates. This unified experimental framework enables direct comparison of DNA methylation effects across chromatin scales while minimizing confounding variables such as DNA sequence, salt concentration, and experimental setup, which have varied across previous studies and likely contributed to discrepancies in the literature.

Specifically, we show that CpG methylation**:**

i) Increases naked DNA stiffness. These results are consistent with several previous studies reporting methylation-induced stiffening of DNA (refs. 29, 35–39), although they differ from the findings of Zaichuk and Marko, 2021 (DOI: 10.1016/j.bpj.2021.03.039, Ref. 36). These discrepancies likely arise from differences in DNA sequence composition, CpG density and distribution, ionic conditions, and the experimental methods used to probe DNA mechanics. Our study minimizes these variables by examining all chromatin states within a single experimental framework, underscoring the importance of performing measurements under directly comparable experimental conditions.

ii) Decreases nucleosome stability within chromatin fibers. We find that the unwrapping energy per nucleosome is lower in +5mC chromatin (**Fig. 2E**), indicating that DNA methylation facilitates nucleosome unwrapping. This result is consistent with previous experimental and computational studies performed in the context of mono-nucleosomes (Ngo et al. 2016, doi: 10.1038/ncomms10813), yet it provides additional mechanistic insights. First, our experiments measure nucleosome unwrapping within chromatin fibers, which provide a more chromatin-like context than isolated mononucleosomes examined in the previous study. Second, the earlier work relied on site-specific fluorescent reporters monitoring local structural transitions at predefined sites, whereas our experiments directly measure nucleosome mechanical stability in a label-free manner through force-extension relationships. Because the two approaches probe distinct physical observables, our measurements provide an independent and complementary mechanical characterization of nucleosome stability across the full unwrapping process.

In summary, our study provides a systematic mechanical characterization of the effects of DNA methylation across chromatin scales, from free DNA to nucleosomes and chromatin fibers, within a single experimental framework. This integrated approach allows direct comparisons across chromatin states and clarifies how DNA methylation influences chromatin mechanics across multiple levels of organization.

Figures 3, 4, and 5 report on the properties of condensates measured through FRAP and VPT, with and without HP1alpha. There are significant differences attributable to methylation-

though not in all cases; for example, the relaxation times derived from data in Figure 4 show a minor difference that is barely larger than the error bars.

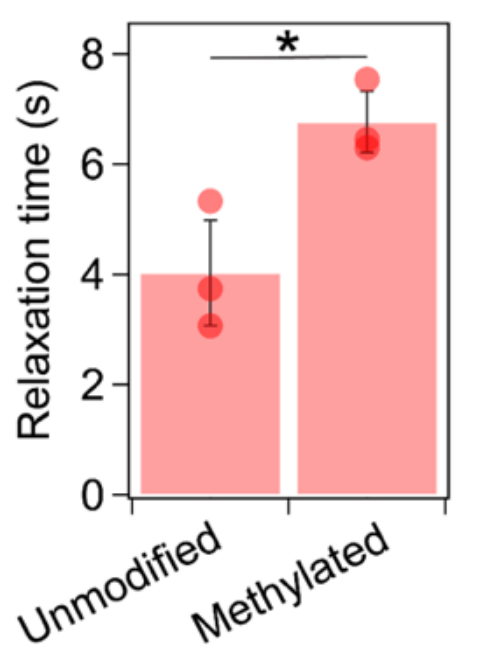

Figure R1. Statistical significance between the relaxation time corresponding to the unmodified (-5mC) and methylated (+5mC) chromatin condensates was determined using a two-sided Student's t-test (*p<0.05). Error bars denote the standard deviation of the mean for n=3 technical replicates, with individual data points overlayed.

The difference between the network relaxation time corresponding to the -5mC (relaxation time = 4.02 ± 0.95 s) and +5mC (relaxation time = 6.76 ± 0.56 s) chromatin condensates is ~1.5-fold and statistically significant (p=0.0124, **Fig. R1**). This shift in the relaxation time reflects strengthening of the interaction network of the chromatin condensates for methylated chromatin condensates.

Importantly, changes of similar magnitude in the network relaxation time have previously been associated with meaningful alterations in condensate material properties[1,2]. In our prior work (*Science Advances* 10.7 (2024): eadi6539, **Fig. R2a-c**), we observed a comparable ~1.5-fold increase in relaxation time (~14 ms to ~20 ms) in synthetic peptide–ssDNA condensates with programmable material properties, which corresponded to a substantial (~2.5-fold) increase in condensate viscosity (**Fig. R2d-e**). These findings demonstrate that ~1.5-fold shift in relaxation time can reflect substantial changes in condensate viscoelasticity and network strength.

In addition, both the MSD measurements and the spatial extent of bead motion indicate significant differences in condensate material properties between the unmethylated and methylated conditions (**Fig 4B, C**). Collectively, these observations support the conclusion

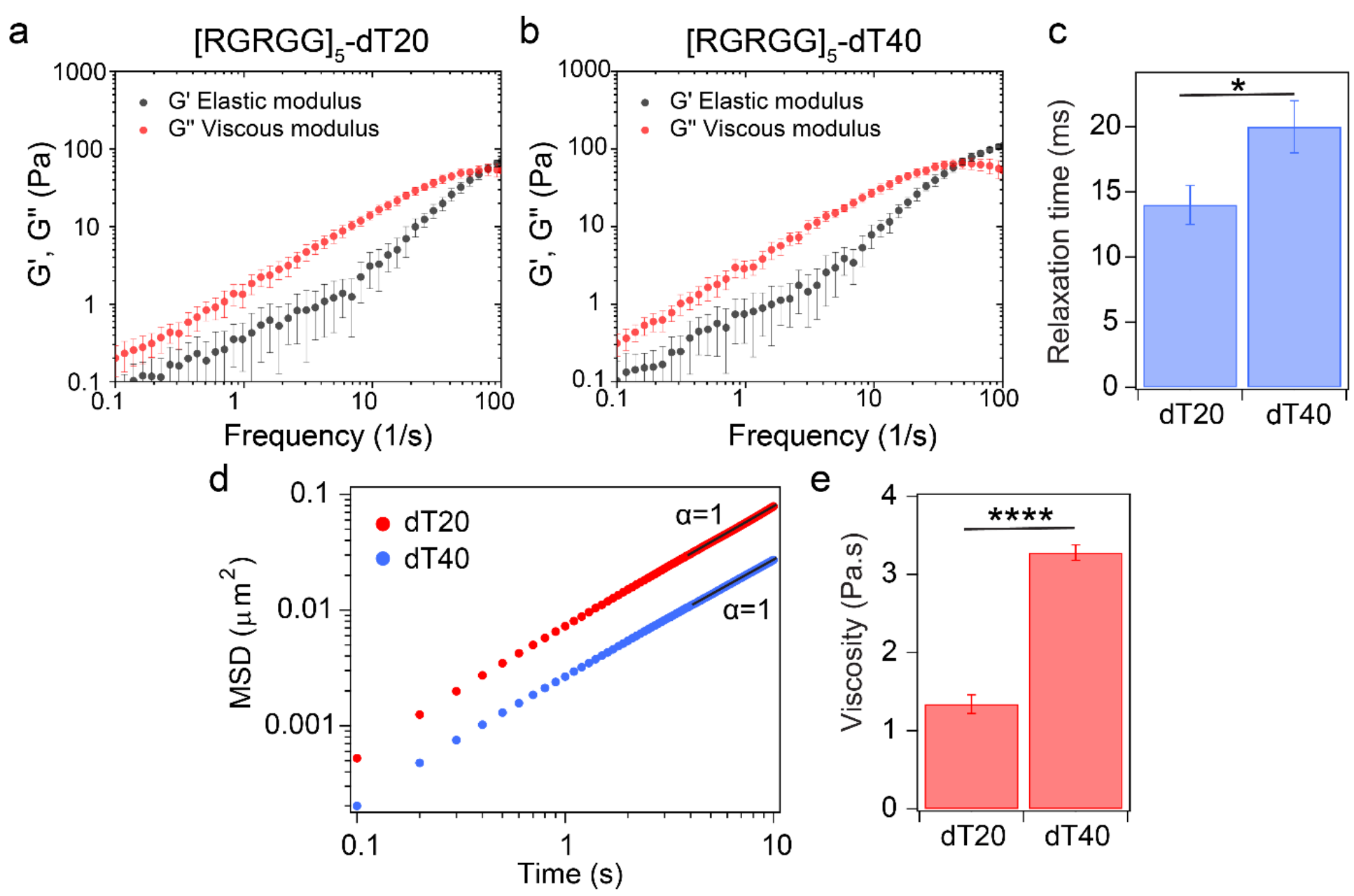

Figure R2. Data reproduced from Alshareedah et al. Science Advances 10.7 (2024): eadi6539 showing (a) complex shear moduli estimated using passive microrheology optical tweezers (pMOT) for synthetic polypeptide-ssDNA condensates– (a) $(RGRGG)_5$–dT20 and (b) $(RGRGG)_5$–dT40. (c) Terminal relaxation time for $(RGRGG)_5$–dT20 and $(RGRGG)_5$–dT40 condensates systems calculated from the inverse of the crossover frequency shown in (a) and (b). (d) Ensemble average mean-squared displacement (MSD) estimated from the motion of 200 nm beads embedded inside the two condensate systems. (e) Viscosity of $(RGRGG)_5$–dT20 and $(RGRGG)_5$–dT40 condensates estimated from the MSD shown in (d). Statistical significance was determined by performing a two-sided Student's t-test (*p<0.05, ****p<0.0001) between the relaxation time and viscosity corresponding to $(RGRGG)_5$–dT20 and $(RGRGG)_5$–dT40 condensates. The analysis revealed a p-value of 0.0113 for the test for relaxation time in (b) and p=0.000015 for viscosity in (e). Error bars denote the standard deviation of the mean for n=3 technical replicates.

that the observed ~1.5-fold shift in relaxation time represents meaningful and statistically significant difference in chromatin condensate material state.

However, what is unsatisfying to me, at least in the current state of the project, is that there is no mechanistic understanding of the phenomena, nor are there functional implications explored here.

We respectfully disagree that the study lacks mechanistic insight and functional implications, and we have revised the abstract and the manuscript to better highlight these aspects.

At the **mechanistic level,** our data show that CpG methylation alters chromatin mesoscale organization through changes in DNA physical properties and nucleosome dynamics. Specifically, the increase in DNA stiffness and nucleosomal DNA unwrapping translates into a more viscoelastic network within chromatin assemblies, indicating altered inter-nucleosome connectivity and/or increased lifetime of nucleosome-nucleosome interactions. This counterintuitive phenomenon, where **nucleosomes that are locally less stable give rise to condensates with increased viscoelasticity**, highlights the importance of distinguishing dynamics occurring within individual nucleosomes (e.g., DNA breathing and nucleosome conformational changes) from inter-nucleosome interactions that occur at the mesoscale within chromatin fibers. Although methylated nucleosomes are individually less mechanically stable, the increased conformational plasticity may facilitate local chromatin rearrangements that promote inter-nucleosome interactions within condensates and reduce large-scale chromatin mobility. This concept is consistent with previous work (Sanulli et al., 2019, doi: 10.1038/s41586-019-1669-2), which showed that nucleosome conformational dynamics facilitate HP1α-mediated chromatin condensation by exposing interaction surfaces on the nucleosome core that promote multivalent inter-nucleosome interactions. Similarly, although individual nucleosomes become mechanically less stable upon DNA methylation, the resulting increase in conformational flexibility may facilitate inter-nucleosome contacts and enhance chromatin network connectivity within condensates. Importantly, DNA methylation does not alter phase boundaries (**Fig. 3B**), indicating that the thermodynamic driving forces for phase separation remain unchanged, while the internal connectivity and relaxation dynamics of the dense phase are altered.

Functionally, these findings indicate that DNA methylation influences chromatin organization by modulating inter-nucleosome interactions that are sensed by chromatin factors such as HP1α. This represents a conceptual shift from the prevailing view that DNA methylation primarily regulates genome function by recruiting methyl-binding proteins or by directly modulating DNA–protein interactions. Instead, our data show that **DNA methylation alters inter-nucleosome interactions and thereby modulates factor binding by reshaping chromatin architecture**. This implies that these mesoscale changes can influence the recruitment of factors that do not contain DNA methylation–binding domains, such as HP1α. These mechanistic and conceptual implications are now more prominently discussed in the manuscript (lines 258-278, and 329-346).

In addition, we have included new data demonstrating: (1) concentration-dependent effects of CpG methylation (**Fig. S5C**), and (2) synergistic effects between CpG methylation and H3K9 methylation in promoting HP1α-mediated chromatin condensation (**Fig. 5B, C**), further strengthening the mechanistic framework of the study.

Although I applaud the authors for their multipronged experimentation, and as much as I appreciate the general idea that methylation can change the properties of condensates, I am not sure EMBO J is the right venue for this work.

We believe this work is suited for EMBO Reports because it provides a mechanistic physical basis linking DNA methylation to heterochromatin organization. By showing that CpG methylation fundamentally alters the material properties of chromatin condensates, which are sensed by HP1α, our work describes an additional layer through which DNA methylation can regulate chromatin organization beyond canonical methyl-binding protein recruitment.

I also found the inclusion and discussion of their cryoET data unconvincing. They claim that there is a difference due to methylation but show only images without any quantification; consequently, it is unclear how readers are supposed to judge the validity of their claim.

We agree with the reviewer that the cryoET images do not provide quantitative information about chromatin organization between the samples. Rigorous quantification of the cryoET datasets is challenging due to the high density and overlap of nucleosomes within chromatin condensates, currently preventing reliable particle identification and segmentation using available computational approaches. Although we explored preliminary coarse quantification approaches based on global density features (**Fig. R3**), these analyses rely on simplifying assumptions and therefore do not provide sufficiently robust metrics to support quantitative conclusions. For this reason, and because they do not substantially add beyond the conclusions already supported by the FRAP and VPT measurements, we chose to present the cryoET data as qualitative observations that provide visual structural context for the chromatin condensates analyzed in this study, illustrating the overall architecture and chromatin density within the condensates. In the revised manuscript, we further toned down the interpretation of the cryoET data, shown in the Supplementary **Fig. S4**, to explicitly indicate that these images are intended to provide structural context rather than quantitative analysis.

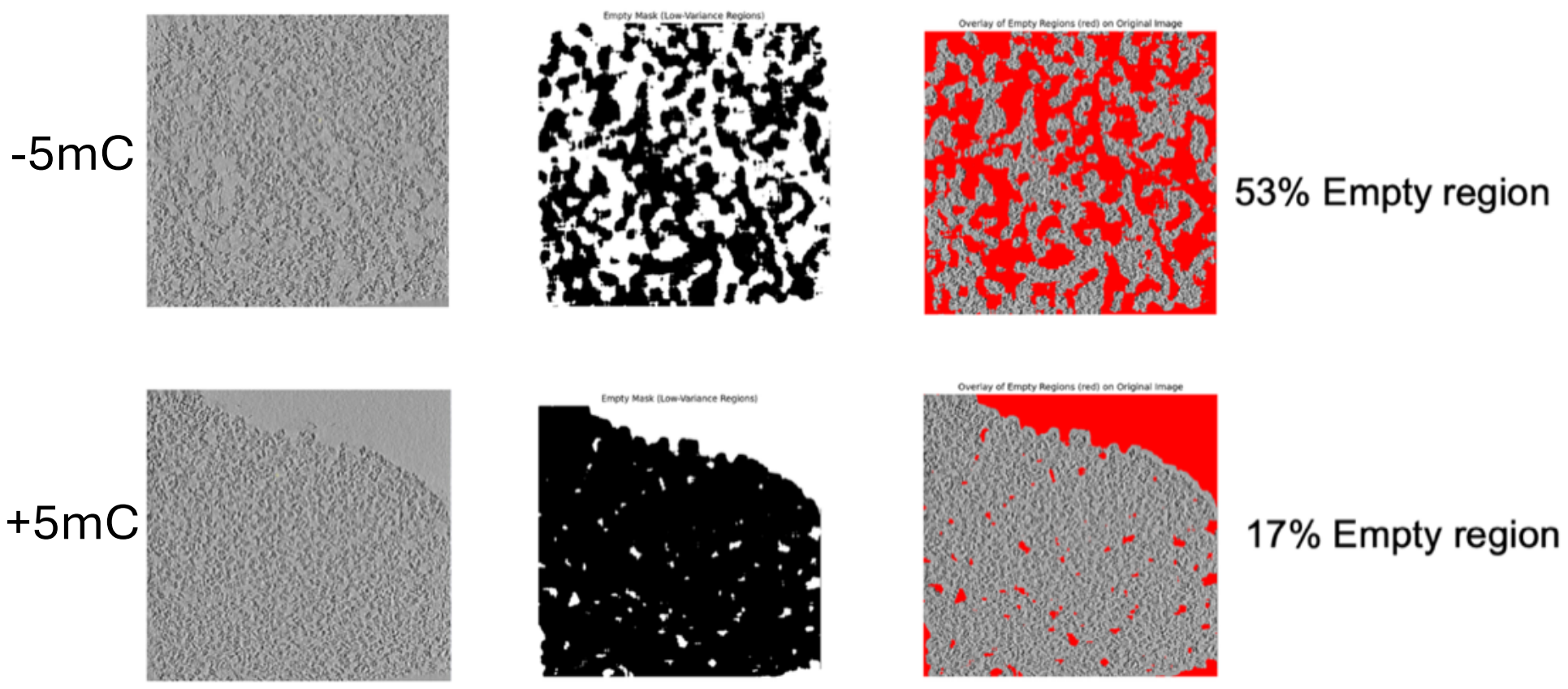

Figure R3: Manual threshold-based segmentation and quantification of low-density regions in condensate reconstructions. Representative cryo-ET slices were analyzed in Python using manually selected intensity and variance thresholds to segment low-density regions corresponding to empty or solvent-rich voids. For each example, the original tomographic slice is shown on the left, the thresholded binary mask of low-density regions is shown in the middle, and the segmented regions overlaid on the original image are shown on the right. The upper sample contains a larger fraction of low-density space, with segmented regions occupying 53% of the analyzed area, whereas the lower sample shows a denser internal network with only 17% low-density regions.

Referee #2:

Referee Report for Daris et al. "Multiscale Physical Effects of CpG Methylation on DNA Mechanics, Nucleosome Wrapping, and Chromatin Condensates" The authors investigate the role of DNA methylation of CpG dinucleotides, all the way from DNA mechanics to nucleosome stability, to chromatin condensates. A strength of the work is that it combines a range of different methods and investigates the effect of methylation at different scales and levels of molecular complexity. The authors conclude that DNA modification can act via multiscale mechanics and not only by directing enzymes / biochemical signaling. In some places, the conclusions could perhaps be discussed more clearly and/or more critically?

We have expanded the results and their discussion to more clearly articulated the implications of these findings across multiple scales of chromatin organization. In addition, the Abstract has been revised to better reflect these conceptual advances and the broader significance of the study.

Specific points of attention are outlined below.

- I am a little puzzled by the reported increase in DNA bending persistence length, in particular given that Zaichuk and Marko, using similar methods, found a clear decrease in Lp (from 47 down to 32 nm after methylation)?

Indeed, our results differ from the findings of Zaichuk and Marko but are consistent with several previous experimental and computational studies reporting methylation-induced stiffening of DNA (refs. 29, 34, 37–40). Notably, these studies employed different DNA sequences and buffer conditions, which likely contribute to the discrepancies between reports. The effects of CpG methylation are sequence dependent because the number and distribution of methylated sites vary between constructs, and buffer composition is also known to influence DNA mechanical properties (Wang et al. 1997, doi: 10.1016/S0006-3495(97)78780-0; Manning GS. 2015, doi: 10.1002/bip.22585; Punkkinen et al. 2005, doi: 10.1529/biophysj.105.063099; Smith et al. 1992, doi: 10.1126/science.1439819). Because direct comparison across chromatin scales was central to our study, we used the same DNA sequence for both naked DNA and nucleosome array experiments and performed all measurements in the same physiologically relevant buffer conditions. This point is now clarified in lines 119-123 and 182.

- The authors report that methylation the stability of assembled nucleosomes and makes them easier to disrupt. It then seems contradictory that methylation would decrease chromatin dynamics and lead to "denser and more viscoelastic" chromatin condensates? If nucleosomes are easier to disrupt, wouldn't that lead to more dynamics and looser condensates? I am not doubting the experimental findings, I am just looking for a better intuitive explanation.

We thank the reviewer for this insightful comment. This observation, while initially counterintuitive, represents one of the key findings of our study, as it highlights the importance of distinguishing dynamics occurring within individual nucleosomes from inter-nucleosome interactions that occur at the mesoscale within chromatin condensates.

Specifically, we find that increased nucleosomal DNA unwrapping induced by DNA methylation promotes nucleosome–nucleosome interactions within chromatin fibers, thereby strengthening the condensate network. This concept is consistent with previous work (Sanulli et al., 2019, doi: 10.1038/s41586-019-1669-2), which showed that nucleosome dynamics facilitate HP1α- and salt-mediated chromatin condensation by exposing interaction surfaces on the nucleosome core that promote multivalent inter-nucleosome interactions. Thus, although individual nucleosomes become mechanically less stable upon DNA methylation, the resulting increase in conformational plasticity may facilitate local chromatin rearrangements and inter-nucleosome contacts while reducing large-scale chromatin mobility. Together, these effects can promote denser chromatin assemblies with increased viscoelasticity and internal friction within the condensate. We have expanded on this point in the revised manuscript (Lines 217-221 and 264-278).

- Along the same lines, the statement "The resulting decrease in wrapping stability could facilitate additional nucleosome loading and compaction" (Line 297) seems at odds with a reduced binding energy for nucleosomes upon methylation?

We acknowledge that it may initially appear counterintuitive that the increased persistence length of methylated DNA could promote nucleosome formation. However, this effect can be explained by a reduction in the entropic penalty associated with wrapping methylated DNA, which can outweigh the energetic cost of bending a stiffer polymer. This mechanism is consistent with prior theoretical and experimental studies (Nathan et al. 2002, doi: 10.1006/jmbi.2001.5247; Anselmi et al. 2000, doi: 10.1016/S0006-3495(00)76319-3; Anselmi

et al. 1998, doi:10.1006/jmbi.1998.2575). Thus, CpG methylation can lower the energetic barrier for nucleosome formation and increase the propensity for nucleosome loading along the DNA. Importantly, an increased propensity for nucleosome formation does not necessarily translate into greater mechanical stability of individual nucleosomes, which is consistent with our observations of reduced nucleosome unwrapping stability. We now clarify this point in the revised manuscript (Lines 167-170).

- How is the "low force" regime (e.g. annotated in Figure 2B) defined?

The definition of the “low force” regime in single-molecule studies varies depending on the experimental context and it typically ranges from 0 to 10 pN. In **Fig. 2B**, the “low force” highlighted regime is meant for illustrative purposes. In our quantifications such as in **Fig. 2D**, we specifically define the low force regime as 0.5-5 pN. This clarification has now been added to the revised manuscript (Line 173).

- Minor point: Two figures are called "Figure 1".

Referee #3:

In this paper, Daris and colleagues examine how CpG methylation modulates DNA and chromatin nanomechanics, addressing a topic on which the literature has been controversial and showing effects in different directions depending on the experimental system. Here, the authors use a combination of single-molecule force spectroscopy, FRAP, and nanorheology as well as phase separation assays to determine 5mC dependent chromatin modulation. They show that 5mC increases both the contour length and persistence length of DNA, yielding longer and stiffer duplexes, while at the nucleosome level it weakens histone-DNA interactions and promotes unwrapping at lower forces.

They then turn to phase separation properties and show that methylated chromatin undergoes salt-induced condensation similarly to unmethylated chromatin. However, it exhibits reduced internal mobility as measured by FRAP and increased viscoelasticity with longer relaxation times by nanorheology. Consistent with these altered material properties, CpG methylation greatly changes behavior in presence of HP1alpha, lowering the protein concentration required to drive chromatin condensation.

Overall this is an interesting study showing intriguing effects of chromatin methylation on domain formation. While there is quite some literature no effects of 5mC on DNA and chromatin mechanics, the effects on chromatin condensate formation are not well explored. Here, the authors find indeed that chromatin is less dynamic and condenses significantly easier with HP1alpha. This is very interesting to a broad readership, but I feel like this finding has to be further corroborated/more mechanistic information is needed.

We thank the reviewer for acknowledging the potential interest of our work to a broad readership.

1. The authors report a fourfold reduction in critical HP1alpha concentration required for chromatin condensate formation, when chromatin is methylated at CpGs. To make this more robust, the experiment should be repeated / quantified, and ideally a proper phase diagram should be generated. E.g. with one axis - degree of methylation, and the other axis - concentration of HP1alpha.

The experiments shown are representative of at least three independent replicates, and the threshold concentration is used to quantify the HP1α concentration at which condensates first become detectable by microscopy. These measurements consistently show an approximately four-fold reduction in the HP1α concentration required for assembly formation in the presence of CpG methylation.

To further strengthen this point, we have generated a phase diagram in which both DNA methylation levels and HP1α concentration are varied. This experiment, now included in **Figure S5A**, systematically assesses how CpG methylation shifts the phase boundary for HP1α-driven chromatin condensation.

It would also be helpful if the authors provide sequence information, and localize CpGs, so the reader gets an idea how many sites are implicated.

The 601 repeats with 25 bp linkers contain 13 methylation sites each, all located within the 147 bp 601 core sequence. The DNA construct used for force spectroscopy experiments contains 434 CpG sites in total. A more detailed description of CpG content and distribution is now included in Supplementary **Fig. S1A**.

2. Mechanistically the authors test the effect of free DNA on HP1alpha condensate formation and don't see a difference. They then state that HP1alpha - DNA interactions are not altered. I am not sure that this can be concluded. DNA binding could be directly tested using EMSA. It is also a bit strange, that HP1alpha condensation occurs around the same concentrations in the presence of naked DNA, as with chromatin containing the HP1 binding site H3K9me3. The authors do not give chromatin or DNA concentration used in these experiments either, making this difficult to interpret.

To more directly assess HP1α–DNA interactions, we performed EMSA experiments (**Fig. S5E**) showing that HP1α binds methylated and unmethylated DNA comparably under these conditions, supporting the conclusion that CpG methylation does not substantially alter the HP1α affinity for DNA.

In the original Figure 5BC (now shown in **Fig. S5C**), chromatin concentration was 20 nM, whereas naked DNA experiments were performed at 40 nM. We have now repeated all experiments at 40 nM chromatin/DNA using bright-field inverted microscopy. These data are now shown in **Fig. 5B-D**, and the concentrations are explicitly stated in the figure legends. HP1α phase separates with naked DNA at 2-fold higher concentrations (10 µM) than with chromatin (5 uM), as previously reported (Sanulli et al., 2019, doi: 10.1038/s41586-019-1669-2), although the resulting droplets are typically larger.

3. To further understand what is driving these differences, the authors could compare HP1 hinge mutants (which they purified according to the methods), or chromatin with and without H3K9me3.

This would further allow to pinpoint the origin of the effect.

The HP1α hinge mutants do not form condensates with chromatin, as shown in **Figure R4** from our recent work (Wong et al. 2025 doi: https://doi.org/10.1101/2025.09.21.676939), and therefore would not provide additional mechanistic insight into the origin of the observed effects. We also note that these mutants were erroneously included in the Methods section and have now been removed in the revised manuscript.

Figure for referee with unpublished data and its description has been removed upon request by the authors.

To clarify the contribution of the H3K9me–HP1α interaction, we assessed the effect of CpG methylation in the absence of H3K9me (WT octamers). While CpG methylation reduced the Cc for phase separation ~4 fold in the presence of the H3K9me, the effects

are much milder with WT octamers, indicating that these two features cooperate to promote chromatin compaction.

4. While the authors provide QC information on chromatin fibers for the mechanistic studies in the beginning of the paper, no information is found on chromatin assembled with H3Kc9me3 used in the HP1 assays. Generally, I am convinced that chromatin quality is critical for all these experiments, and differences in array assembly easily override effects based on DNA methylation.

The quality of all chromatin preparations was assessed by native gel electrophoresis prior to downstream experiments. We now provide additional chromatin quality control gels in **Figure S5D**.

5. The authors state that more nucleosomes are detected per fiber on 5mC chromatin for the force spectroscopy experiments. This is surprising, but could explain some of the later findings. E.g. could this increase of nucleosome loading (through whatever mechanism, maybe indicated by the somewhat stronger higher-running band in S3) also be responsible for the observed differences dynamics within droplets, i.e. dynamics and different viscoelasticity?

The increased nucleosome loading observed in the force spectroscopy experiments arises from the different DNA constructs used in those assays. Specifically, these experiments employ longer DNA handles (2.0 and 2.7 kb) lacking 601 nucleosome positioning sequences, which can allow additional nucleosome assembly along the DNA.

In contrast, the DNA constructs used for condensate experiments contain only defined 601 positioning sequences spaced by 25 bp linkers, thereby preventing over-assembly and ensuring regular nucleosome positioning. Under the optimized assembly conditions used here, native PAGE analyses show comparable assembly quality between methylated and unmethylated chromatin fibers. Thus, the observed differences in condensate dynamics cannot be attributed to increased nucleosome loading or oversaturation. We have clarified these distinctions between constructs and experimental conditions in the revised manuscript (**Fig. S1A**, Lines 199-201).

6. The authors discuss their cryoET findings in a qualitative way, and provide a few images for justification, which show the claimed differences. But, of course, this is highly subjective, and possibly strongly dependent on the analyzed region, grids etc. In the absence of any quantification over a large area etc. this is really purely for illustrative purposes and no claims towards "open and porous architecture" etc. should be made.

We agree with the reviewer that the cryoET images are primarily illustrative. Rigorous quantification of these datasets remains challenging because the high density of nucleosomes within condensates currently prevents reliable segmentation and particle identification across large regions. Examples of preliminary masking-based analyses are provided in **Fig. R3**; however, these analyses rely on simplifying assumptions and do not provide sufficiently robust quantitative metrics. For this reason, and because they do not substantially add beyond the conclusions already supported by the FRAP and VPT measurements, we chose to present the cryoET data as qualitative observations in the Supplementary Information. We believe the images provide visual structural context for the chromatin condensates analyzed in this study, illustrating the overall architecture and chromatin density within the condensates.

Accordingly, we removed strong claims regarding condensate architecture and explicitly state that the cryoET images are intended to provide qualitative structural context in support of the quantitative FRAP and VPT-based nano-rheology measurements.

Minor points:

7. Fig. 2C: "nucleosome number" is a bit misleading as we do not know which nucleosome is unwrapping. Rather, whichever nucleosome has the lowest stability is unwrapping first, without any spatial order. Maybe label the x-axis "unwrapping event" or something similar.

We have incorporated this in the revised manuscript.

8. Fig 2C: I don't doubt the conclusions. But the statistical testing, i.e. using different tests within the same graph, seems flawed: Although the authors justify the use of whichever tests by pointwise normality testing, the t- and U-tests assess different null hypotheses. A unified statistical analysis across the whole dataset is preferable. Also, the authors show SEM, but no n is given.

To unify the statistical analyses, we now report Whitney-Mann U-tests across the entire dataset.

The n-values for the unmethylated samples are as follows for each unwrapping event: 26, 26, 26, 26, 26, 26, 26, 24, 16, 11, 6, 5. For methylated samples: 37, 37, 37, 37, 37, 37, 35, 35, 35, 32, 26, 20. We felt that including all values directly in the figure caption would reduce readability, but all underlying data remain fully available to the reader in the source data files.

Dear Serena,

Thank you for the submission of your revised manuscript. We have now received the enclosed reports from the referees that were asked to assess it and I am happy to say that both support the publication of your ms now.

Only a few editorial requests will need to be addressed before we can proceed with the official acceptance of your manuscript:

- With your 5 main figures, we will publish your study as a short report. Please correct the Results subheading to "Results and Discussion".

- Please correct the conflict of interest subheading to "Disclosure and Competing Interests Statement" (DCIS)

- Please note that the format of the author list in the ms needs to be the same as in our online submission system: First Name Last Name. There is a last name discrepancy: Rizvi A in the ms vs Aoon Rizvu in the system.

- The AFFILIATION #7 seems to be a company; employment in a biotech company should be stated in DCIS.

- The author credits need to be removed from the ms file. All credits need to be entered during online ms submission.

- The REFERENCE format is not correct: it needs to be alphabetical, not numerical; et al needs to be used after 10 author names; Bibliography should be renamed to References. Please correct.

- In the author checklist, 3 responses have not been selected, this must be corrected. Please also answer all questions on the statistics and send us a new, completed checklist.

- The Propel postdoctoral fellowship is missing as a separate funder in our online system, please add.

- The callouts for the suppl. figures need to be corrected. The correct nomenclature for the supporting figures should be Figure EV1, etc. and needs to be corrected in all places; "Supplementary Figures" should be changed to "Expanded View Figure Legends".

- The Source Data (SD) need to be uploaded as one zip folder per figure. The SD checklist needs to be uploaded as a Related Ms file.

- Materials and Methods should be just Methods

- The manuscript sections should be in the following order: Title page - Abstract - Introduction - Results - Discussion - Methods - Data Availability - Acknowledgments - Disclosure Statement & Competing Interests - References - Figure Legends - (Main Tables with legends if applicable) - Expanded View Figure Legends.

- The Abbreviations section needs to be removed from the manuscript. Abbreviations should be defined in brackets after their first mention in the text, not in a list of abbreviations.

- Corresponding author section on p16 needs to be removed from the ms file.

- Our routine image analyses of to be accepted ms detected several potential cell re-uses between Figure 5B and Figure S5C that are not listed in the figure legend. Can you please clarify? IF the same images are re-used deliberately this must be explained in the figure legend.

* Figure Legends - Comments *

- 1. Please note that the exact p values are not provided in the legends of 1C,D; 2C,E,F; S2-D. Please provide exact values as reasonable.
- Please note that the box plots need to be defined in terms of minima, maxima, centre, bounds of box and whiskers, and percentile in the legends of figures 1C,D; 2E,F; 3E; S2-D,E.
- Please note that information related to n is missing in the legends 3E.

EMBO press papers are accompanied online by A) a short (1-2 sentences) summary of the findings and their significance, B) 2-3 bullet points highlighting key results and C) a synopsis image that is exactly 550 pixels wide and 300 pixels high. The synopsis image should provide a sketch of the major findings, like a graphical abstract. Please note that text needs to be readable at the

final size. Please send us this information along with the final manuscript.

I look forward to seeing a final version of your manuscript as soon as possible.

Best wishes,
Esther

Esther Schnapp, PhD
Senior Editor
EMBO reports

Referee #1:

I the revised paper the authors have addressed all reviewer comments. They have improved the mechanistic discussion and functional consequences of their results. They have provided further data to underpin their analysis and weakened some of their claims, in particular with respect to the cryoEM analysis, which still nicely illustrates their findings.
Together, I think this is a well performed and interesting study and I support its publication in Embo Reports.

Referee #2:

I feel that the authors have addressed my previous concerns adequately during the revision and that the manscript is now ready for publication.

All minor editorial requests have been addressed by the authors.

Prof. SERENA SANULLI
Stanford University
Genetics
1291 Welch Road, Room 4400
BioMedical Innovation Building
Stanford, CA 94305
United States

Dear Serena,

I am very pleased to accept your manuscript for publication in the next available issue of EMBO reports. Thank you for your contribution to our journal.

Your manuscript will be processed for publication by EMBO Press. It will be copy edited and you will receive page proofs prior to publication. Please note that you will be contacted by Springer Nature Author Services to complete licensing and payment information.

You may qualify for financial assistance for your publication charges - either via a Springer Nature fully open access agreement or an EMBO initiative. Check your eligibility: https://link.springer.com/journal/44319/how-to-publish-with-us

Should you be planning a Press Release on your article, please notify the production team via this form in order to coordinate publication and release dates: https://forms.cloud.microsoft/e/eH4A8JVP6g

If you have any questions, please do not hesitate to contact the Editorial Office. Thank you for your contribution to EMBO Reports.

Best regards,
Esther

Esther Schnapp, PhD
Senior Editor
EMBO reports

>>> Please note that it is EMBO Reports policy for the transcript of the editorial process (containing referee reports and your response letter) to be published as an online supplement to each paper. If you do NOT want this, you will need to inform the Editorial Office via email immediately. More information is available here: https://link.springer.com/partners/embo-press/editorial-policies#Peer%20review

EMBO Press Author Checklist

Corresponding Author Name: Serena Sanulli and Jorine M Eeftens	
Journal Submitted to: EMBO Reports	
Manuscript Number: EMBOR-2026-63912V2	

USEFUL LINKS FOR COMPLETING THIS FORM
The EMBO Journal - Author Guidelines
EMBO Reports - Author Guidelines
Molecular Systems Biology - Author Guidelines
EMBO Molecular Medicine - Author Guidelines

Reporting Checklist for Life Science Articles (updated January
This checklist is adapted from Materials Design Analysis Reporting (MDAR) Checklist for Authors. MDAR establishes a minimum set of requirements in transparent reporting in the life sciences (see Statement of Task: 10.31222/osf.io/9sm4x). Please follow the journal's guidelines in preparing your
Please note that a copy of this checklist will be published alongside your article.

Abridged guidelines for figures
1. Data
The data shown in figures should satisfy the following conditions:
- ➞ the data were obtained and processed according to the field's best practice and are presented to reflect the results of the experiments in an accurate and unbiased manner.
- ➞ ideally, figure panels should include only measurements that are directly comparable to each other and obtained with the same assay.
- ➞ plots include clearly labeled error bars for independent experiments and sample sizes. Unless justified, error bars should not be shown for technical
- ➞ if n<5, the individual data points from each experiment should be plotted. Any statistical test employed should be justified.
- ➞ Source Data should be included to report the data underlying figures according to the guidelines set out in the authorship guidelines on Data

2. Captions
Each figure caption should contain the following information, for each panel where they are relevant:
- ➞ a specification of the experimental system investigated (eg cell line, species name).
- ➞ the assay(s) and method(s) used to carry out the reported observations and measurements.
- ➞ an explicit mention of the biological and chemical entity(ies) that are being measured.
- ➞ an explicit mention of the biological and chemical entity(ies) that are altered/varied/perturbed in a controlled manner.
- ➞ the exact sample size (n) for each experimental group/condition, given as a number, not a range;
- ➞ a description of the sample collection allowing the reader to understand whether the samples represent technical or biological replicates (including how many animals, litters, cultures, etc.).
- ➞ a statement of how many times the experiment shown was independently replicated in the laboratory.
- ➞ definitions of statistical methods and measures:
  - common tests, such as t-test (please specify whether paired vs. unpaired), simple χ2 tests, Wilcoxon and Mann-Whitney tests, can be unambiguously identified by name only, but more complex techniques should be described in the methods section;
  - are tests one-sided or two-sided?
  - are there adjustments for multiple comparisons?
  - exact statistical test results, e.g., P values = x but not P values < x;
  - definition of 'center values' as median or average;
  - definition of error bars as s.d. or s.e.m.

Please complete ALL of the questions below.
Select "Not Applicable" only when the requested information is not relevant for your study.

Materials

Newly Created Materials	Information included in the manuscript?	In which section is the information available? (Reagents and Tools Table, Materials and Methods, Figures, Data Availability Section)
New materials and reagents need to be available; do any restrictions apply?	Not Applicable	

Antibodies	Information included in the manuscript?	In which section is the information available? (Reagents and Tools Table, Materials and Methods, Figures, Data Availability Section)
For **antibodies** provide the following information:<br>- Commercial antibodies: RRID (if possible) or supplier name, catalogue number and or/clone number<br>- Non-commercial: RRID or citation	Not Applicable	

DNA and RNA sequences	Information included in the manuscript?	In which section is the information available? (Reagents and Tools Table, Materials and Methods, Figures, Data Availability Section)
**Short novel DNA or RNA including primers, probes**: provide the sequences.	Not Applicable	

Cell materials	Information included in the manuscript?	In which section is the information available? (Reagents and Tools Table, Materials and Methods, Figures, Data Availability Section)
**Cell lines:** Provide species information, strain. Provide accession number in repository **OR** supplier name, catalog number, clone number, and/**OR** RRID.	Not Applicable	
**Primary cultures:** Provide species, strain, sex of origin, genetic modification status.	Not Applicable	
Report if the cell lines were recently **authenticated** (e.g., by STR profiling) and tested for mycoplasma contamination.	Not Applicable	

Experimental animals	Information included in the manuscript?	In which section is the information available? (Reagents and Tools Table, Materials and Methods, Figures, Data Availability Section)
**Laboratory animals or Model organisms:** Provide species, strain, sex, age, genetic modification status. Provide accession number in repository **OR** supplier name, catalog number, clone number, **OR** RRID.	Not Applicable	
**Animal observed in or captured from the field:** Provide species, sex, and age where possible.	Not Applicable	
Please detail **housing and husbandry conditions**.	Not Applicable	

Plants and microbes	Information included in the manuscript?	In which section is the information available? (Reagents and Tools Table, Materials and Methods, Figures, Data Availability Section)
**Plants:** provide species and strain, ecotype and cultivar where relevant, unique accession number if available, and source (including location for collected wild specimens).	Not Applicable	
**Microbes:** provide species and strain, unique accession number if available, and source.	Not Applicable	

Human research participants	Information included in the manuscript?	In which section is the information available? (Reagents and Tools Table, Materials and Methods, Figures, Data Availability Section)
If collected and within the bounds of privacy constraints report on age, sex and gender or ethnicity for all study participants.	Not Applicable	

Core facilities	Information included in the manuscript?	In which section is the information available? (Reagents and Tools Table, Materials and Methods, Figures, Data Availability Section)
If your work benefited from core facilities, was their service mentioned in the acknowledgments section?	Not Applicable	

Design

Study protocol	Information included in the manuscript?	In which section is the information available? (Reagents and Tools Table, Materials and Methods, Figures, Data Availability Section)
If study protocol has been **pre-registered, provide DOI in the manuscript**. For clinical trials, provide the trial registration number **OR** cite DOI.	Not Applicable	
Report the **clinical trial registration number** (at ClinicalTrials.gov or equivalent), where applicable.	Not Applicable	

Laboratory protocol	Information included in the manuscript?	In which section is the information available? (Reagents and Tools Table, Materials and Methods, Figures, Data Availability Section)
Provide DOI OR other citation details if **external detailed step-by-step protocols** are available.	Not Applicable	

Experimental study design and statistics	Information included in the manuscript?	In which section is the information available? (Reagents and Tools Table, Materials and Methods, Figures, Data Availability Section)
Include a statement about **sample size** estimate even if no statistical methods were used.	Yes	Figure captions, methods, source data.
Were any steps taken to minimize the effects of subjective bias when allocating animals/samples to treatment (e.g. **randomization procedure**)? If yes, have they been described?	Yes	Methods.
Include a statement about **blinding** even if no blinding was done.	Yes	Methods.
Describe **inclusion/exclusion criteria** if samples or animals were excluded from the analysis. Were the criteria pre-established?<br><br>If sample or data points were omitted from analysis, report if this was due to attrition or intentional exclusion and provide justification.	Yes	This applies to inclusion of data for unwrapping events and can be found in the methods.
For every figure, are **statistical tests** justified as appropriate? Do the data meet the assumptions of the tests (e.g., normal distribution)? Describe any methods used to assess it. Is there an estimate of variation within each group of data? Is the variance similar between the groups that are being statistically compared?	Yes	Figures, Materials ans Methods

Sample definition and in-laboratory replication	Information included in the manuscript?	In which section is the information available? (Reagents and Tools Table, Materials and Methods, Figures, Data Availability Section)
In the figure legends: state number of times the experiment was **replicated** in laboratory.	Yes	Figures
In the figure legends: define whether data describe **technical or biological replicates**.	Yes	Figures

Ethics

Ethics	Information included in the manuscript?	In which section is the information available? (Reagents and Tools Table, Materials and Methods, Figures, Data Availability Section)
Studies involving **human participants**: State details of **authority granting ethics approval** (IRB or equivalent committee(s), provide reference number for approval.	Not Applicable	
Studies involving **human participants**: Include a statement confirming that **informed consent** was obtained from all subjects and that the experiments conformed to the principles set out in the WMA Declaration of Helsinki and the Department of Health and Human Services Belmont Report.	Not Applicable	
Studies involving **human participants:** For publication of **patient photos**, include a statement confirming that consent to publish was obtained.	Not Applicable	
Studies involving experimental **animals**: State details of **authority granting ethics approval** (IRB or equivalent committee(s), provide reference number for approval. Include a statement of compliance with ethical regulations.	Not Applicable	
Studies involving **specimen and field samples:** State if relevant **permits** obtained, provide details of authority approving study; if none were required, explain why.	Not Applicable	

Dual Use Research of Concern (DURC)	Information included in the manuscript?	In which section is the information available? (Reagents and Tools Table, Materials and Methods, Figures, Data Availability Section)
Could your study fall under dual use research restrictions? Please check biosecurity documents and list of **select agents and toxins** (CDC): https://www.selectagents.gov/sat/list.htm	Not Applicable	
If you used a select agent, is the security level of the lab appropriate and reported in the manuscript?	Not Applicable	
If a study is subject to dual use research of concern regulations, is the name of the **authority granting approval and reference number** for the regulatory approval provided in the manuscript?	Not Applicable	

Reporting

The MDAR framework recommends adoption of discipline-specific guidelines, established and endorsed through community initiatives. Journals have their own policy about requiring specific guidelines and recommendations to complement MDAR.

Adherence to community standards	Information included in the manuscript?	In which section is the information available? (Reagents and Tools Table, Materials and Methods, Figures, Data Availability Section)
State if relevant guidelines or checklists (e.g., **ICMJE, MIBBI, ARRIVE, PRISMA**) have been followed or provided.	Not Applicable	
For **tumor marker prognostic studies,** we recommend that you follow the **REMARK** reporting guidelines (see link list at top right). See author guidelines, under 'Reporting Guidelines'. Please confirm you have followed these guidelines.	Not Applicable	
For **phase II and III randomized controlled trials**, please refer to the **CONSORT** flow diagram (see link list at top right) and submit the CONSORT checklist (see link list at top right) with your submission. See author guidelines, under 'Reporting Guidelines'. Please confirm you have submitted this list.	Not Applicable	

Data Availability

Data availability	Information included in the manuscript?	In which section is the information available? (Reagents and Tools Table, Materials and Methods, Figures, Data Availability Section)
Have **primary datasets** been deposited according to the journal's guidelines (see 'Data Deposition' section) and the respective accession numbers provided in the Data Availability Section?	Not Applicable	
Were **human clinical and genomic datasets** deposited in a public access-controlled repository in accordance to ethical obligations to the patients and to the applicable consent agreement?	Not Applicable	
Are **computational models** that are central and integral to a study available without restrictions in a machine-readable form? Were the relevant accession numbers or links provided?	Not Applicable	
If publicly available data were reused, provide the respective **data citations in the reference list**.	Not Applicable	
