# Supplementary material for: Multiscale physical effects of CpG methylation on DNA mechanics, nucleosome wrapping, and chromatin condensates
Source: EMBO Rep. 2026 Sep 3;27(19):5449–67. doi: 10.1038/s44319-026-00894-2 (PMC13649787; doi:10.1038/s44319-026-00894-2)
Supplement: Supplementary file 2 [file 44319_2026_894_MOESM7_ESM.pdf]

Expanded View Figures

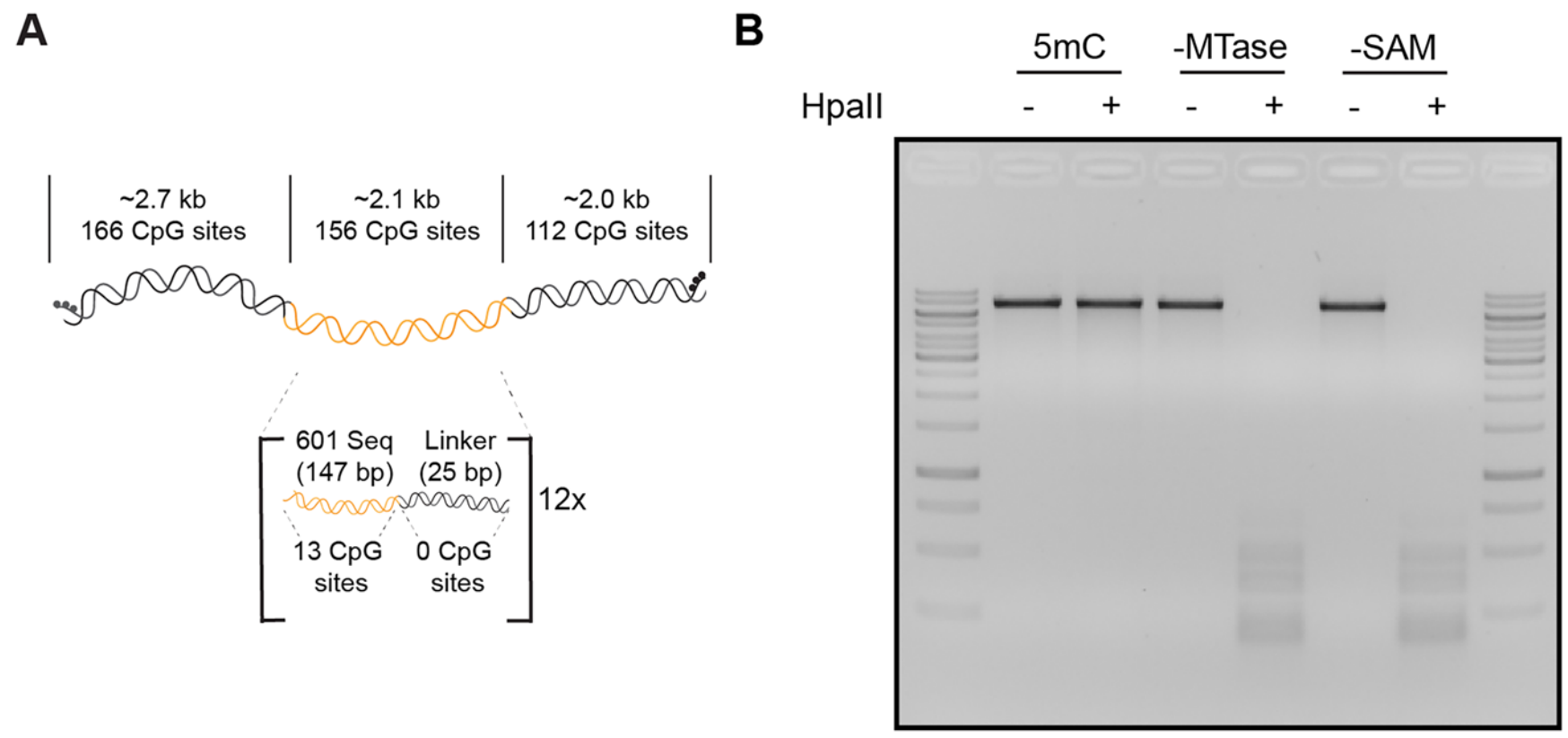

Figure EV1. Verification of CpG methylation of the DNA construct.

(**A**) The CpG methyltransferase (MTase) M.SssI was used to methylate all cytosines at CpG sites within the DNA construct. The number of CpG methylation sites present within each DNA feature, including the Widom 601-positioning sequences, linker DNA, and flanking DNA handles, is indicated. (**B**) Digestion with the methylation-sensitive restriction enzyme HpaII confirmed complete methylation: the DNA was protected from cleavage following MTase treatment (lane 3). In the absence of MTase or SAM, the DNA remained sensitive to HpaII digestion (lanes 5 and 7). The fully methylated DNA construct was used for force spectroscopy and as the template for chromatin reconstitution.

 © The Author(s)

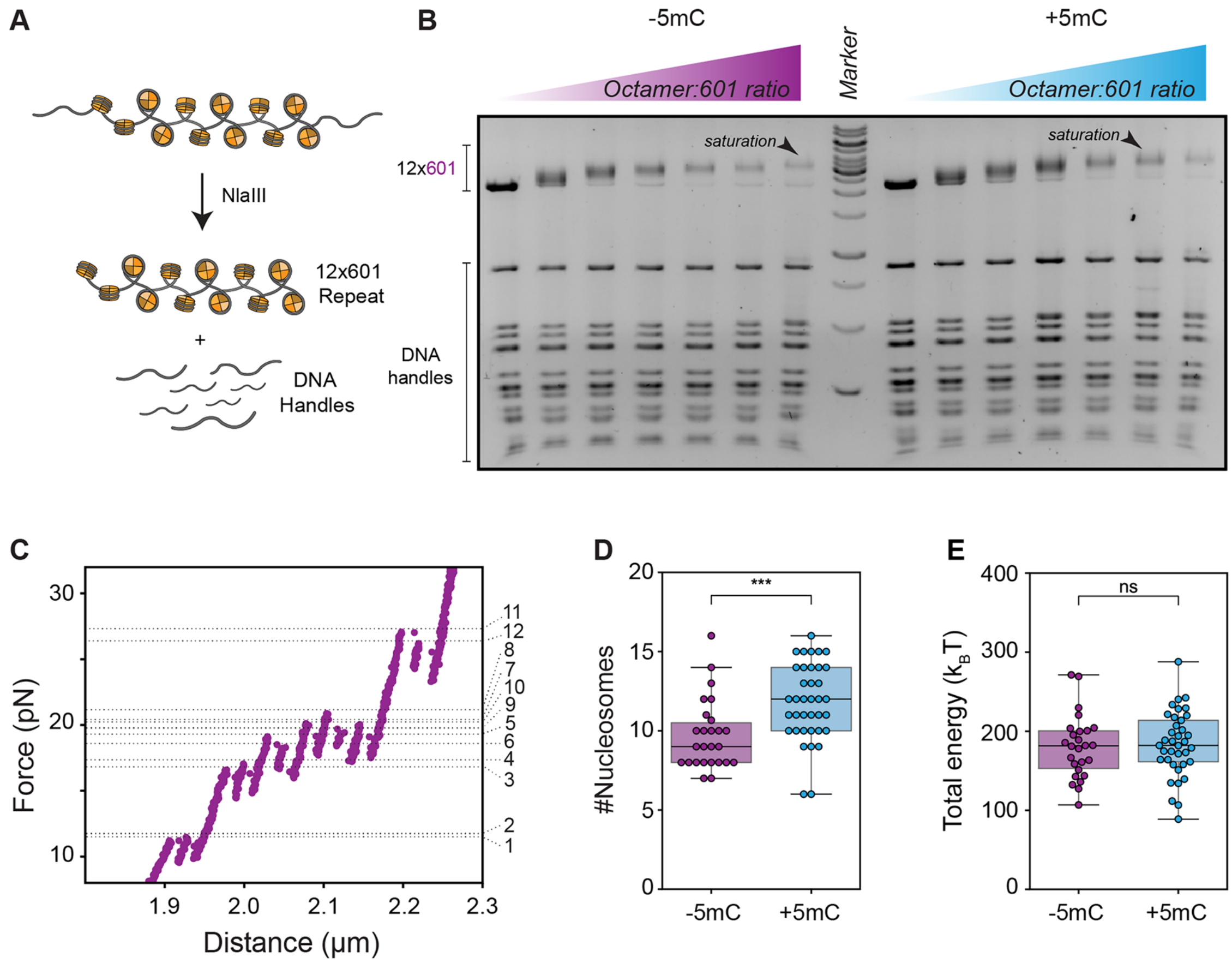

Figure EV2. Determining the number of nucleosomes per chromatin fiber.

(A) Enzymatic validation of nucleosome assembly. Reconstituted chromatin fibers were digested with NlaIII to distinguish between nucleosome assembly at the 601 sequences and the assembly on the DNA handles. **(B)** Saturation of nucleosome assembly determined by EMSA. Chromatin fibers were reconstituted with increasing octamer:DNA molar ratios to ensure full saturation of the 601 sites. NlaIII digestion releases the 12×601 segment (upper bands) and further digests the DNA handles (residual lower bands). Progressive mobility shifts of the 601 reflect increasing nucleosome occupancy until saturation, whereas excess octamer results in additional shifts corresponding to over-assembly on the DNA handles. **(C)** Discrete unwrapping steps in the high-force regime correspond to inner turn rupture events, enabling quantification of nucleosome number per tether. Peak forces before each rupture event were used to identify individual unwrapping transitions. **(D)** Quantification of rupture events shows a significantly higher number of nucleosomes per chromatin fiber in the +5mC condition (Mann–Whitney *U* test: $P = 0.00039$, $n = 37$ and $n = 26$ for +5mC and −5mC, respectively). Box plots are composed of a box from the 25th to 75th percentile with the median as a line and whiskers calculated via the Tukey method. **(E)** Total stretching energy (area under the curve in the low-force regime) is not significantly different between −5mC and +5mC fibers (Student's *t* test: ns, $P = 0.7189$, $n = 37$ and $n = 26$ for +5mC and −5mC, respectively). This apparent similarity results from increased nucleosome occupancy in the 5mC condition, rather than unchanged fiber mechanics. Box plots are composed of a box from the 25th to 75th percentile with the median as a line and whiskers calculated via the Tukey method.

© The Author(s)

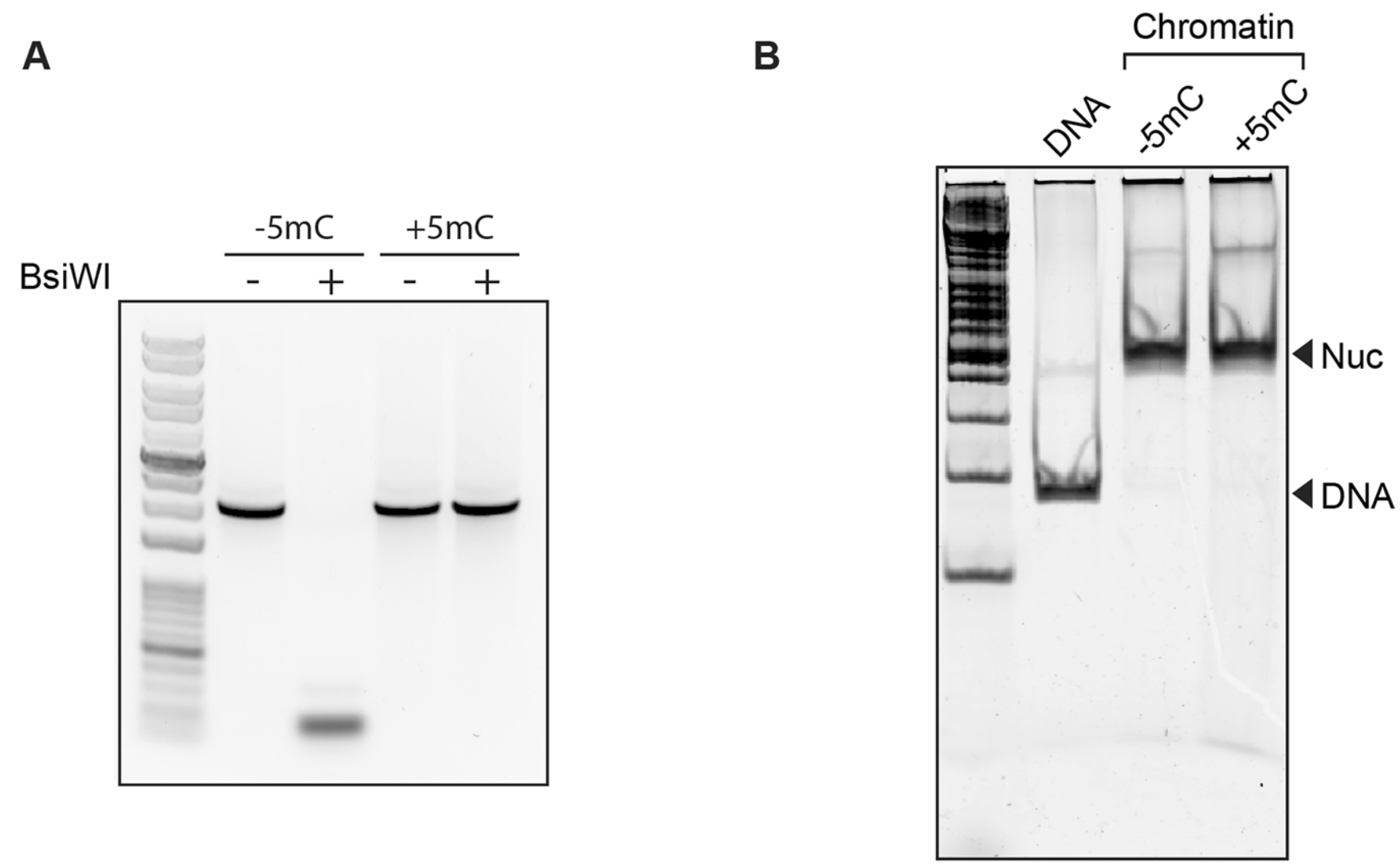

Figure EV3. Chromatin quality control.

(**A**) Digestion with the methylation-sensitive restriction enzyme BsiWI confirmed complete methylation. DNA was protected from cleavage following treatment with MTase and SAM. The methylated DNA construct was used as the template for chromatin reconstitution. (**B**) Enzymatic validation of chromatin assembly. Naked dsDNA and reconstituted chromatin fibers were digested with BstXI and analyzed on a native gel. Both −5mC and +5mC chromatin show complete and comparable nucleosome occupancy.

© The Author(s)

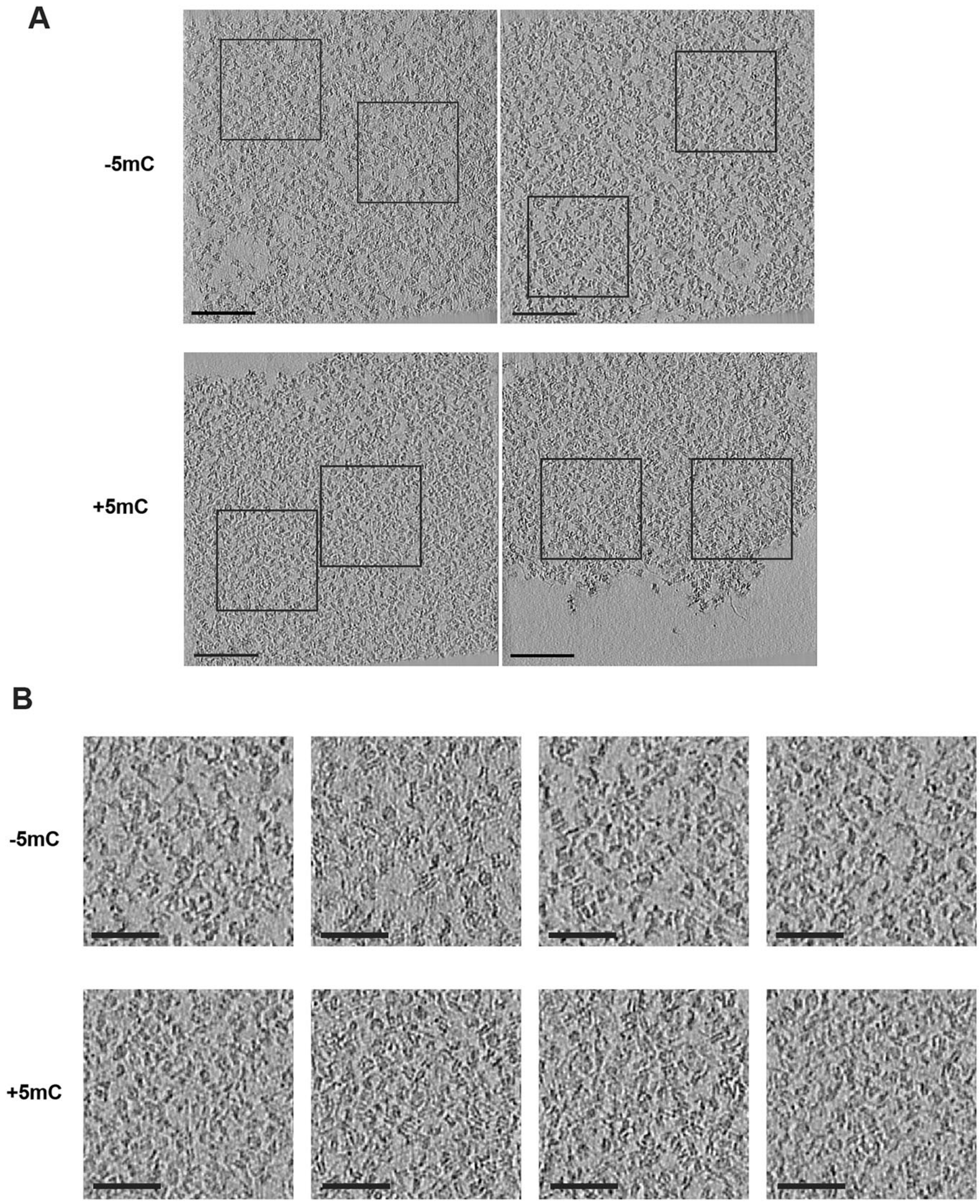

Figure EV4. Qualitative assessment of chromatin condensates by Cryo-ET.

(**A**) Representative enhanced views generated by summing five consecutive tomogram slices of −5mC (top) and +5mC (bottom) chromatin condensate. (**B**) Magnified views of the regions indicated by black squares in (**A**), highlighting differences in internal organization. Scale bar = 50 nm.

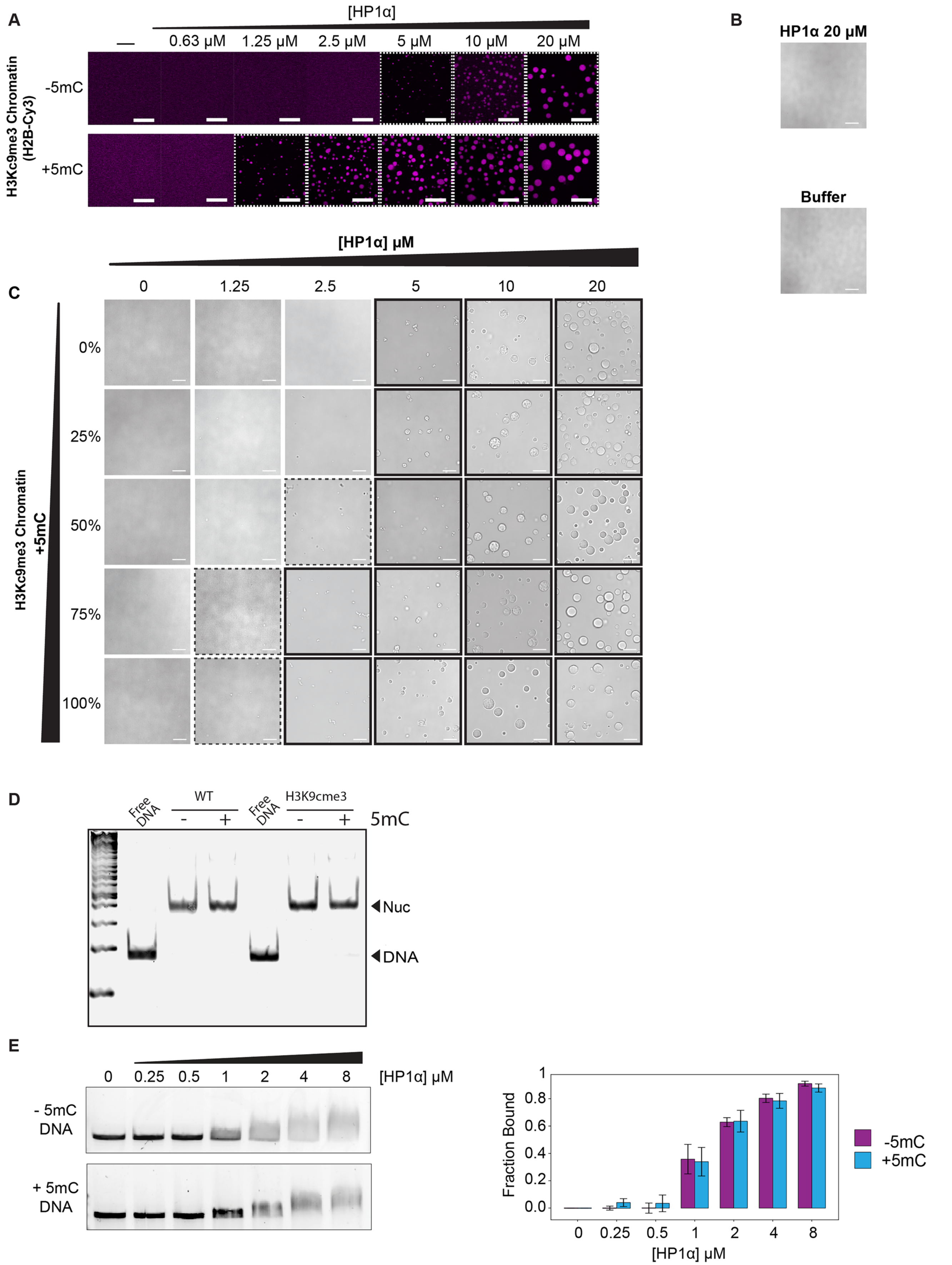
A
[HP1α]
—
0.63 µM
1.25 µM
2.5 µM
5 µM
10 µM
20 µM
H3Kc9me3 Chromatin (H2B-Cy3)
-5mC
+5mC
B
HP1α 20 µM
Buffer
C
[HP1α] µM
0
1.25
2.5
5
10
20
H3Kc9me3 Chromatin +5mC
0%
25%
50%
75%
100%
D
Free DNA
WT
Free DNA
H3K9cme3
- +
- +
5mC
Nuc
DNA
E
0
0.25
0.5
1
2
4
8
[HP1α] µM
- 5mC DNA
+ 5mC DNA
Fraction Bound
1
0.8
0.6
0.4
0.2
0.0
0
0.25
0.5
1
2
4
8
[HP1α] µM
-5mC
+5mC

 © The Author(s)

Figure EV5. HP1α interactions with chromatin and DNA.

(**A**) Confocal images of the indicated H3Kc9me3 chromatin condensates formed at increasing HP1α concentrations. Chromatin (40 nM) is fluorescently labeled. White dashed squares indicate conditions in which condensates are observed. (**B**) HP1α and buffer-only controls showing no phase separation. Scale bar = 4 µm. (**C**) Phase diagram showing HP1α-driven phase separation across varying HP1α concentrations and chromatin CpG methylation levels. Chromatin fibers contain H3Kc9me3 nucleosomes (40 nM). Black squares mark conditions in which condensates are observed, dotted lines indicate conditions in which small irregular assemblies are observed. The 0% and 100% methylation conditions correspond to those shown in Fig. 5B. Scale bar = 4 µm. (**D**) Enzymatic validation of chromatin assembly, showing complete and comparable nucleosome occupancy. Naked dsDNA and reconstituted chromatin fibers were digested with BstXI and analyzed on a native gel. (**E**) EMSA comparing HP1α binding to methylated and unmethylated dsDNA (12 × 601), showing that CpG methylation does not substantially alter HP1α–DNA interaction. Left: representative native gels; right: quantification of the fraction bound from three independent replicates. Mean ± SEM.

© The Author(s)
